# Add-on effects of total glucosides of paeony on conventional therapies for psoriasis: a systematic review and meta-analysis of randomized controlled trials

**DOI:** 10.3389/fphar.2025.1527288

**Published:** 2025-02-19

**Authors:** Ziqing Li, Jie Lu, Kewen Guan, Haiwen Liang, Chuanjian Lu, Jingjie Yu

**Affiliations:** ^1^ The Second Clinical School of Guangzhou University of Chinese Medicine, Guangzhou, Guangdong, China; ^2^ Guangdong Provincial Hospital of Chinese Medicine and Guangdong Provincial Academy of Chinese Medical Sciences, The Second Affiliated Hospital of Guangzhou University of Chinese Medicine, Guangzhou, Guangdong, China; ^3^ State Key Laboratory of Dampness Syndrome of Chinese Medicine, Guangdong-Hong Kong-Macau Joint Lab on Chinese Medicine and Immune Disease Research and Guangdong Provincial Key Laboratory of Clinical Research on Chinese Medicine Syndrome, Guangzhou, Guangdong, China

**Keywords:** total glucosides of paeony, conventional therapies, psoriasis, effect, meta-analysis

## Abstract

**Background:**

Psoriasis is an inflammatory and recurrent dermatological disease that is associated with multiple comorbidities. Conventional psoriasis therapies such as acitretin capsule and narrow-band ultraviolet B radiation (NB-UVB) are prone to decreased efficacy and adverse events in long-term application. Total glucosides of paeony (TGP), a plant extract from Radix Paeoniae Alba, are commonly used in conjunction with conventional therapies for psoriasis. This study aims to elucidate the add-on effect of TGP on conventional therapies in the treatment of psoriasis.

**Methods:**

Seven databases were searched from their inception to March 2024. Randomized controlled trials (RCTs) using TGP in conjunction with conventional therapies for psoriasis were included. The Risk of Bias 2.0 (RoB 2.0) tool was used to assess bias risk, and data analysis was conducted using RevMan V.5.4. Evaluation outcomes mainly involved a 60% or greater reduction of Psoriasis Area and Severity Index score (PASI 60) and a 50% or greater reduction of Psoriasis Area and Severity Index score (PASI 50).

**Results:**

This meta-analysis ultimately included 36 RCTs with 3,140 participants. The findings indicated that TGP combined with conventional therapies were superior to conventional therapies used alone on PASI 60 (RR = 1.32, 95% CI: 1.25 to 1.39, *P* < 0.00001) and PASI 50 (RR = 1.44, 95% CI: 1.13 to 1.84, *P* = 0.004). Several types of conventional therapies were prone to PASI 60 response when combined with TGP than conventional therapies using alone, such as oral medication (RR = 1.40, 95% CI: 1.14, to 1.71, *P* = 0.001), topical medication (RR = 1.47, 95% CI: 1.24 to 1.74, *P* < 0.00001), and NB-UVB (RR = 1.29, 95% CI: 1.16 to 1.43, *P* < 0.00001). Furthermore, the results suggested that TGP might reduce the incidence of adverse events occurred by conventional therapies for psoriasis.

**Conclusion:**

This meta-analysis demonstrated the preliminary clinical evidence supporting the addition of TGP to conventional therapies in treating psoriasis. Owing to the limited methodological quality of the included studies, well-designed RCTs are required to further illustrate the add-on effect of TGP on conventional therapies for psoriasis.

**Systematic Review Registration:**

https://www.crd.york.ac.uk/PROSPERO/display_record.php?RecordID=439904, identifier CRD42023439904.

## 1 Background

Psoriasis is a chronic, recurrent, inflammatory dermatological disease, with a global prevalence of approximately 125 million (WHO, 2016; Griffiths et al., 2021; Psoriasis Committee, Dermatology and Venereology Branch, Chinese Medical Association, 2023). Patients with psoriasis are probably susceptible to serious comorbidities such as malignancy, coronary atherosclerosis, and psoriatic arthritis (Amin et al., 2020; Boehncke et al., 2010; Kommoss et al., 2023; Mayor, 2016). Apprehensively, during the long-term and recrudescent course, patients with psoriasis are at risk of substantial negative impacts on psychological health, such as depression and suicidal behaviors (Bardazzi et al., 2022; Bell et al., 2021). Nowadays, published guidelines for psoriasis declare that conventional therapies applied commonly in clinical practice contain oral systemic treatment, topical medication, biological agents, and phototherapy (Nast et al., 2018; Nast et al., 2020; Psoriasis Committee, Dermatology and Venereology Branch, Chinese Medical Association, 2023). Given the reduced efficacy and increased adverse events in long-term application (Chia et al., 2023; Thatiparthi et al., 2022; Katz et al., 1999), conventional therapies, as a type of psoriasis treatment, face challenges in optimizing therapeutic strategies.

Total glucosides of paeony (TGP), a natural extract from Radix Paeoniae Alba, have been recommended by the guideline as an alternative therapy for the treatment of psoriasis (Psoriasis Committee, Dermatology and Venereology Branch, Chinese Medical Association, 2023). Published research studies have indicated that TGP has an immunological effect on anti-inflammation and immune regulation in psoriasis (Lei et al., 2023; Li et al., 2019). Moreover, relevant clinical studies have illustrated the potential of TGP in improving psoriatic symptoms and reducing common adverse events (Yu et al., 2017; Zheng et al., 2019). Expectantly, combining application with TGP is conducive to be a novel optimized therapeutic strategy for conventional therapies in treating psoriasis. However, to date, there is a lack of solid clinical evidence to evaluate the add-on effect of TGP on conventional therapies and support the combination of TGP with conventional therapies as a novel therapeutic option in the treatment of psoriasis.

Thus, it is essential to conduct a systematic review and meta-analysis in accordance with the standard of Cochrane Handbook to elucidate the add-on effect of TGP on conventional therapies for the treatment of psoriasis. This study has the potential to identify a novel optimized therapeutic strategy for conventional therapies in treating psoriasis and provide instructive insights for further investigation.

## 2 Methods

This systematic review and meta-analysis was undertaken following the standard guidance of the Preferred Reporting Items for Systematic Review and Meta-Analysis (PRISMA). In addition, the registration of this study was completed on the Prospective Register of Systematic Reviews (CRD42023439904).

### 2.1 Search strategy

This meta-analysis searched for randomized controlled trials (RCTs) comparing TGP to conventional therapies in the treatment of PV from database inception to March 2024. Databases included three English databases, namely, PubMed, Embase, and Web of science, and four Chinese databases, namely, China National Knowledge Infrastructure (CNKI), China Biomedical Literature Database (CBM), Chinese Scientific Journal Database (VIP), and Wanfang database (Wanfang).

### 2.2 Eligibility criteria

The inclusion and exclusion criteria were cooperatively established by two researchers (CL and JY).

#### 2.2.1 Inclusion criteria

The inclusion criteria were presented as follows: 1) patients diagnosed with psoriasis and without limitations on gender, age, race, economic status, or education; 2) treatment of TGP associated with conventional therapies (such as acitretin capsule and methotrexate) applied in the intervention group, while conventional therapies used in the control group; 3) outcomes concerning the Psoriasis Area and Severity Index (PASI) score or adverse events; and 4) study designed with RCTs.

#### 2.2.2 Exclusion criteria

The exclusion criteria were presented as follows: 1) studies with incomplete data or data errors; 2) duplicate publication; 3) reviews, consensus-based studies, commentaries, conference abstracts, case reports, and animal or cell experiments; and 4) inadequate outcomes for systematic review and meta-analysis.

### 2.3 Study selection and data extraction

Two researchers (ZL and KG) first screened the titles and abstracts of the included studies and then removed the duplicates. Next, full-text screening was simultaneously undertaken, and any conflicts were determined by the third researcher (JY) through the discussion. Accordingly, two researchers (ZL and KG) independently extracted the detailed characteristics of the included studies, such as age, gender, intervention, comparator, administration and dosage, and adverse events. The collated data were cross-checked by another two researchers (JL and HL).

### 2.4 Quality assessment

The bias risk of the included studies was separately evaluated by two researchers (JL and HL) using the Risk of Bias 2.0 (RoB 2.0) tool according to the Cochrane Handbook V.6.5. The six domains of bias risk were presented as follows: 1) randomization process; 2) deviations from intended interventions; 3) missing outcome data; 4) measurement of the outcome; 5) selection of the reported result; and 6) overall. The risk bias of every domain was classified as low, some concern, or high. Response to every signaling question included five options: yes (Y), probably yes (PY), probably no (PN), no (N), and no information (NI). Any discrepancies were discussed and resolved by all researchers.

### 2.5 Statistical analysis

Review Manager version 5.4 and Stata version 17.0 were utilized to analyze the effect of TGP associated with conventional therapies for psoriasis. The enumeration data were represented by relative risk (RR) for each effect quantity. Relatively, the measurement data were presented with both the mean difference (MD) and the 95% confidence interval (95% CI). The data pooling model for meta-analysis was chosen based on the presence and magnitude of heterogeneity. The heterogeneity of the included data was evaluated using the chi-squared (X^2^) test and the I-squared (*I* ^*2*^) test. The fixed-effects model was selected when there was acceptable heterogeneity of the findings (*I* ^*2*^ < 50%). Otherwise, the random effects model was used when there was statistically significant heterogeneity among detected findings (*I* ^*2*^ ≥ 50%). Additionally, the random effects model was also applied for meta-analysis when there was apparent heterogeneity among the studies. Sensitivity analysis was conducted to assess the stability of the conclusion, and meta-regression analyses were performed to identify the source of heterogeneity. Subgroup analysis was conducted based on the outcome measures, treatment duration, and adverse events. *p* < 0.05 was considered statistically significant. The publication bias of the included studies was evaluated using a funnel plot and Egger’s test.

## 3 Results

### 3.1 Search results

**FIGURE 1:**
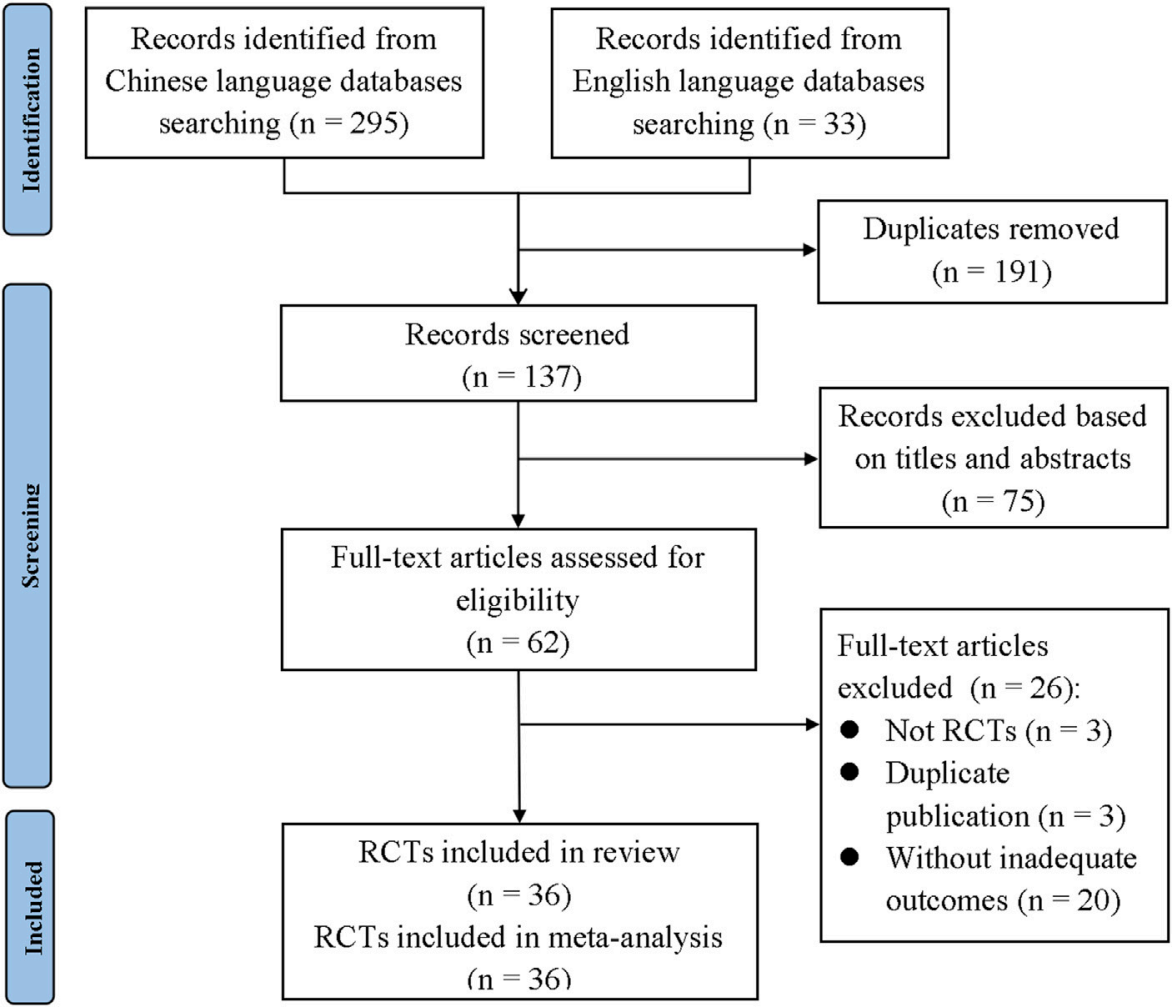
PRISMA flowchart of the search process. TPG, total glucosides of paeony; RCT, randomized controlled trial.

A total of 328 studies were retrieved according to the eligibility criteria. After eliminating the duplicates, 137 studies were screened with their titles and abstracts. Then, the remaining 62 studies that met the inclusion criteria were given access to the full text. A total of 26 studies were excluded for the following reasons: 1) not RCTs; 2) duplicate publication; and 3) without inadequate outcomes. Finally, 36 studies were included in this meta-analysis (Yu et al., 2017; Cai et al., 2014; Chen et al., 2014; Ding, 2020; Du et al., 2017; Du et al., 2012; Gao, 2020; Guo and Xu, 2019; Guo, 2016; He et al., 2014; He et al., 2012; Hu et al., 2012a; Hu et al., 2012b; Hua et al., 2012; Huang, 2015; Huang and Huang, 2011; Jiang et al., 2014; Jiang et al., 2013; Li, 2017; Li et al., 2016; Lin, 2017; Liu et al., 2014; Liu and Wang, 2011; Lv, 2020; Pang, 2011; Shang et al., 2020; Song et al., 2017; Wang, 2016; Yang et al., 2013; Zhang et al., 2015; Zhang et al., 2016; Zhang et al., 2022; Zhang and Wang, 2018; Zhang et al., 2011; Zhu, 2016; Zou et al., 2015). The detailed flowchart of the searching process is shown in Figure 1.

### 3.2 Study characteristics

A total of 36 RCTs were included, with 3,140 participants. Concerning the study design, all studies used a two-arm parallel design, with the exception of five studies (Huang, 2015; Liu et al., 2014; Liu and Wang, 2011; Zhang et al., 2011; Zhu, 2016; Zou et al., 2015) using three-arm parallel design and one study (Zhu, 2016) using the four-arm parallel design. Among the included studies, five studies (Hua et al., 2012; Jiang et al., 2013; Liu et al., 2014; Pang, 2011) reported the stage of psoriasis, and eight studies (Yu et al., 2017; Chen et al., 2014; He et al., 2014; Huang, 2015; Jiang et al., 2014; Lin, 2017; Zhang et al., 2015; Zhang et al., 2022) indicated the severity of psoriasis. During the study period, seven studies (Yu et al., 2017; Cai et al., 2014; Du et al., 2012; Jiang et al., 2014; Zhang et al., 2016; Zhang et al., 2022; Zou et al., 2015) received 12-week treatment, while five studies (Hu et al., 2012a; Jiang et al., 2013; Liu et al., 2014; Zhang et al., 2011; Zhu, 2016) received 6-week treatment. The remaining studies used an 8-week treatment period. Only four studies (Liu et al., 2014; Yang et al., 2013; Zhang and Wang, 2018; Zou et al., 2015) mentioned the follow-up period. Concerning the treatment applied in the included studies, all the intervention groups were treated with TGP and conventional therapies, while conventional therapies were applied alone in the control group. In the included studies, acitretin capsules, narrow-band ultraviolet B radiation (NB-UVB), and topical ointment were the most conventional therapies. The detailed characteristics of the included studies are presented in Table 1. Regarding the dosage of TGP, a dose of 0.6 g was used in all studies except one study (Huang, 2015) that use 0.4 g. Concerning the administration of TGP, a majority of studies administered TGP thrice per day, while five studies (Ding, 2020; Guo and Xu, 2019; Hua et al., 2012; Lv, 2020; Zhu, 2016) applied it twice daily. The details of dosage and administration are shown in Table 2.

**TABLE 1:** Characteristics of included studies.

First author, year	Country; setting	Blinding; number of arms	Treatment and follow-up duration	Type of psoriasis	Severity of psoriasis	Duration of psoriasis: mean (SD)	No. of I/C; dropouts	Age: mean (SD); gender: M/F	Outcome measure
Zhang et al. (2022)	China; hospital inpatients	NS; 2	12 w; 0	Psoriasis vulgaris	Severe	I: 16.7 (3.2) m C: 16.8 (3.8) m	I: 40/40; 0 C: 40/40; 0	I: 35.21(5.28); 29/11 C: 34.92(5.60); 30/10	⑤⑥
Gao (2020)	China; hospital inpatients	NS; 2	8 w; 0	NS	NS	I: 4.32 (0.72) y C:4.29 (0.63) y	I: 47/47; 0 C: 47/47; 0	I: 43.57(2.04); 26/21 C: 43.92(2.01); 24/23	⑤⑥
Shang et al. (2020)	China; hospital inpatients	NS; 2	8 w; 0	Psoriasis vulgaris	NS	I: 5.37 (0.23) y C: 5.21 (0.18) y	I: 30/30; 0 C: 30/30; 0	I: 34.6(4.2); 17/13 C: 35.7(5.3); 16/14	②⑥
Ding (2020)	China; hospital inpatients	NS; 2	4 w; 0	NS	NS	I: 6.4 (2.6) y C: 6.8 (2.3) y	I: 39/39; 0 C: 39/39; 0	I: 29.2(6.4); 14/25 C: 28.7(6.4); 16/23	⑤
Lv (2020)	China; hospital inpatients	NS; 2	8 w; 0	Psoriasis vulgaris	NS	I: 7.63 (1.83) y C: 7.79 (1.72) y	I: 56/56; 0 C: 56/56; 0	I: 44.58(7.12); 31/25 C: 45.01(6.93); 29/27	②④
Guo and Xu (2019)	China; hospital inpatients	NS; 2	8 w; 0	NS	NS	I: 3.87 (1.72) y C: 4.23 (1.68) y	I: 23/23; 0 C: 23/23; 0	I:44.32(5.86); 14/10 C: 43.28(5.94); 13/10	②⑤⑥
Zhang and Wang. (2018)	China; hospital inpatients	NS; 2	8 w; 24 w	Psoriasis vulgaris	NS	I: 6.74 (1.43) y C: 6.62 (1.40) y	I: 50/50; 0 C: 50/50; 0	I: 44.12(5.77); 35/15 C: 44.19(5.80); 33/17	③⑤
Yu et al. (2017)	China; hospital outpatients	NS; 2	12 w; 0	Psoriasis vulgaris	Moderate and severe	I: 138.62 (120.61) m C: 158.3 (103.12) m	I: 53/40; 13 C: 55/44; 11	I: 38.43(12.06); 35/18 C: 38.11(12.05); 36/19	①③⑥
Song et al. (2017)	China; hospital inpatients	NS; 2	8 w; 0	Psoriasis vulgaris	NS	I: 127.68 (96.75) m C: 129.62 (98.62) m	I: 63/63; 0 C: 63/63; 0	I: 43.52(12.03); 48/15 C: 43.29(11.76); 49/14	②④⑤
Lin (2017)	China; NS	NS; 2	8 w; 0	Psoriasis vulgaris	Moderate	I: 18.81 (5.33) y C: 19.74 (6.21) y	I: 67/67; 0 C: 62/62; 0	I: 39.28(10.21); 39/28 C: 40.18(11.83); 37/25	⑤
Du et al. (2017)	China; hospital outpatients	NS; 2	4 w; 0	Psoriasis vulgaris	NS	NS	I: 32/32; 0 C: 32/32; 0	I: 37.3(5.2); 21/11 C: 32.2(5.4); 18/14	⑥
Li (2017)	China; hospital outpatients	NS; 2	8 w; 0	Psoriasis vulgaris	NS	I: 61.47 (22.53) m C: 62.18 (24.25) m	I: 37/37; 0 C: 37/37; 0	I: 37.15(9.24); 22/15 C: 36.52(9.16); 24/13	②⑥
Li et al. (2016)	China; hospital outpatients	NS; 2	8 w; 0	Psoriasis vulgaris	NS	I: 6.16 (4.29) y C: 7.16 (4.98) y	I: 30/27; 3 C: 30/28; 2	I: 45.23 (15.52); 19/11 C: 42.51 (9.52); 14/16	②
Wang (2016)	China; NS	NS; 2	NS; NS	Psoriasis vulgaris	NS	NS	I: 30/30; 0 C: 30/30; 0	I: 39.87 (5.24); 16/14 C: 41.64 (4.38); 17/13	②
Zhang et al. (2016)	China; hospital inpatients	NS; 2	12 w; 0	Psoriasis vulgaris	NS	I: 13.6 (3.9) y C: 12.4 (3.5) y	I: 48/48; 0 C: 48/48; 0	I: 36.8 (5.3); 30/18 C: 36.2 (5.1); 28/20	②④⑥
Guo (2016)	China; hospital inpatients	NS; 2	8 w; 0	Psoriasis vulgaris	NS	NS	I: 30/30; 0 C: 30/30; 0	I: 36.4 (6.7); 19/11 C: 36.1 (6.4); 18/12	⑥
Zhu (2016)	China; hospital out/inpatients	NS; 4	6 w; 0	Psoriasis vulgaris	NS	NS	I1: 11/11; 0 I2: 11/11; 0 C: 11/11; 0	I1: 35.5 (10.4); 4/7 I2: 34.4 (10.0); 7/4 C: 36.6 (10.2); 7/4	②⑤⑥
Huang (2015)	China; NS	NS; 3	24 w; 0	Psoriasis vulgaris	Moderate and severe	NS	I: 16/16; 0 C: 16/16; 0	35.2 (10.8); 26/22	②⑥
Zou et al. (2015)	China; NS	NS; 3	12 w; 24w	Psoriasis vulgaris	NS	NS	I: 25/25; 0 C: 26/26; 0	37.75 (5.61); 42/34	②⑥
Zhang et al. (2015)	China; NS	NS; 2	24w; 0	Psoriasis vulgaris	Mild to moderate	I: 5.1 y C: 4.9 y	I:48/45; 3 C: 48/46; 2	I: 38.6; 22/26 C: 41.2; 20/28	①⑥
Cai et al. (2014)	China; hospital outpatients	NS; 2	12 w; 0	Psoriasis vulgaris	NS	NS	I: 38/38; 0 C: 35/35; 0	I: 43.66 (13.35); 24/14 C: 45.40 (12.26); 22/13	③⑥
Chen et al. (2014)	China; hospital out/inpatients	NS; 2	8 w; 0	Psoriasis vulgaris	Moderate and severe	I: 6.35 (3.24) y C: 6.86 (4.03) y	I: 30/28; 2 C: 30/27; 3	I: 40.12 (13.23); 16/12 C: 42.35 (12.25); 14/13	②⑥
Jiang et al. (2014)	China; hospital out/inpatients	NS; 2	12 w; 0	Psoriasis vulgaris	Moderate and severe	I: 5.4 (2.3) y C: 5.7 (2.5) y	I: 35/35; 0 C: 34/34; 0	I: 33.9 (12.4); 20/15 C: 34.4 (13.7); 19/15	②⑥
Liu et al. (2014)	China; hospital outpatients	NS; 3	6 w; 12w	Psoriasis vulgaris	NS	I: 6.25 (3.23) y C: 5.89 (2.97) y	I: 36/36; 0 C: 32/32; 0	I: 39.15 (13.68); 18/18 C: 41.71 (12.93); 17/15	②⑥
He et al. (2014)	China; hospital outpatients	NS; 2	8 w; 0	Psoriasis vulgaris	Moderate	NS	I: 78/78; 0 C: 76/76; 0	NS	②⑥
Yang et al. (2013)	China; hospital outpatients	NS; 2	8 w; 12w	Psoriasis vulgaris	NS	I: 69.7 (62.5) m C: 67.4 (63.3) m	I: 34/34; 0 C: 34/34; 0	I: 35.74 (11.53); 18/16 C: 33.89 (12.78); 17/17	②⑥
Jiang et al. (2013)	China; hospital out/inpatients	NS; 2	6 w; 0	Psoriasis vulgaris	NS	15.48 (12.25) y	I: 38/38; 0 C: 24/24; 0	34.33 (16.45); 40/22	②⑥
Hu et al. (2012a)	China; hospital out/inpatients	NS; 2	6 w; 0	Psoriasis vulgaris	NS	I: 11.6 (3.5) y C: 12.3 (2.7) y	I: 50/50; 0 C: 50/50; 0	I: 37.3 (11.5); 27/13 C: 35.8 (12.3); 28/22	②⑤
Du et al. (2012)	China; hospital out/inpatients	NS; 2	12 w; 0	Pustular psoriasis	NS	I: 7 (4.7) y C: 6 (5.1) y	I: 31/31; 0 C: 30/30; 0	I: 41 (5.2); 17/14 C: 43 (6,2); 18/12	②⑥
He et al. (2012)	China; hospital outpatients	NS; 2	8 w; 0	NS	Moderate and severe	I: 5.23 (3.23) y C: 5.09 (3.76) y	I: 32/32; 0 C: 30/30; 0	I: 41.23 (14.17); 18/14 C: 40.18 (11.36); 16/14	②⑥
Hua et al. (2012)	China; hospital outpatients	NS; 2	8 w; 0	Psoriasis vulgaris	NS	I: 42.96 (25.23) m C: 52.65 (11.20) m	I: 23/23; 0 C: 23/23; 0	I: 55.35 (10.92); 16/7 C: 52.65 (11.20); 14/9	②
Hu et al. (2012b)	China; hospital patients (NS)	NS; 2	12 w; 0	Psoriasis vulgaris	NS	I: 3.75y C: 3.75y	I: 90/90; 0 C: 90/90; 0	I: 37.5 (12.2); 48/42 C: 38.5 (11.5); 46/44	②⑥
Zhang et al. (2011)	China; hospital outpatients	NS; 3	6 w; 0	Psoriasis vulgaris	NS	I: 67.3 (59.8) m C: 68.3 (67.2) m	I: 30/30; 0 C: 30/30; 0	I: 35.6 (10.4); 18/12 C: 38.5 (9.8); 16/14	②⑥
Pang (2011)	China; hospital patients (NS)	NS; 2	8 w; 0	Psoriasis vulgaris	NS	I: 8.32 (4.61) y C: 5.26 (3.57) y	I: 38/38; 0 C: 30/30; 0	I: 23.41 (6.18); 17/21 C: 27.41 (7.12); 18/12	②⑥
Huang and Huang (2011)	China; hospital patients (NS)	NS; 2	8 w; 0	Psoriasis vulgaris	NS	I: 3.6 y C: 2.8 y	I: 39/39; 0 C: 32/32; 0	I: 24.8; 25/14 C: 23.5; 23/9	②⑥
Liu and Wang (2011)	China; hospital outpatients	NS; 3	4 w; 0	Psoriasis vulgaris	NS	NS	I: 36/36; 0 C: 26/26; 0	38.1 (9.6); 46/44	②⑥

TGP, total glucosides of paeony; C, control; I, intervention; AC, acitretin capsule; NB-UVB, narrow-band UVB; NS, not stated; PASI, Psoriasis Area and Severity Index; w, weeks; y, years; M, male; F, female; ① PASI 75; ② PASI 60; ③ PASI 50; ④ BSA; ⑤ inflammatory factors; ⑥ adverse events.

**TABLE 2:** Detailed information concerning the intervention/comparators of included studies.

First author, year	Intervention (TGP + conventional therapy)	Comparators (conventional therapy)	TGP preparation type and dosage	Conventional therapy dosage and administration
Zhang et al. (2022)	TGP + etanercept	Etanercept	NS, 0.6 g po tid	25 mg sc. twice a week
Gao (2020)	TGP + AC	Acitretin capsule	Capsule, 0.6 g po bid	25 mg/d as initial oral dosage, adjusted dosage based on severity, and medication tolerance
Shang et al. (2020)	TGP + desonide cream	Desonide cream	Capsule, 0.6 g po tid	Topical use, bid
Ding (2020)	TGP + NB-UVB and tacrolimus ointment	Tacrolimus ointment, NB-UVB	Capsule, 0.6 g po bid	Tacrolimus ointment, topical use, bid; NB-UVB, 3 days in a row
Lv (2020)	TGP + AC + compound flumetasone ointment	Acitretin capsule and compound flumetasone ointment	Capsule, 0.6 g po bid	Acitretin capsule, 25–30 mg/d as initial oral dosage, adjusted dosage based on severity, and medication tolerance. The maximum dose does not exceed 75 mg/d; compound flumetasone ointment, topical use, qd or bid
Guo and Xu (2019)	TGP + erythromycin ointment + sulfur ointment	Erythromycin ointment and sulfur ointment	Capsule, 0.6 g po tid	Erythromycin ointment, topical use, qd; sulfur ointment, topical use, tid
Zhang and Wang. (2018)	TGP + AC + NB-UVB	NB-UVB and acitretin capsule	Capsule, 0.6 g po tid	NB-UVB, three times a week; acitretin capsule, 20 mg/d for body mass> 70 kg
Yu et al. (2017)	TGP + AC	Acitretin capsule	Capsule, 0.6 g po bid in the first week and then adjusted dosage to 0.6 g po tid	A dose of 20 mg/day if the patient weighed less than or equal to 70 kg or 30 mg/day if the patient weighed greater than 70 kg
Song et al. (2017)	TGP + AC + compound flumetasone ointment	Acitretin capsule and compound flumetasone ointment	Capsule, 0.6 g po tid	Acitretin capsule, 10 mg/d po; compound flumetasone ointment, qd
Lin (2017)	TGP + NB-UVB + calcipotriol cream	NB-UVB and calcipotriol cream	Capsule, 0.6 g po tid	NB-UVB, three times a week; calcipotriol cream, topical use, bid
Du et al. (2017)	TGP + calcipotriol betamethasone ointment	Calcipotriol betamethasone ointment	Capsule, 0.6 g po tid	Topical use, qd
Li (2017)	TGP + NB-UVB	NB-UVB	Capsule, 0.6 g po tid	qod
Li et al. (2016)	TGP + NB-UVB	NB-UVB	Capsule, 0.6 g po tid	Three times a week
Wang (2016)	TGP + compound flumetasone ointment	Compound flumetasone ointment	Capsule, 0.6 g po tid	Topical use, qd or bid
Zhang et al. (2016)	TGP + methotrexate tablet + vaseline	Methotrexate tablet and vaseline	Capsule, 0.6 g po tid	Methotrexate tablet, 2.5 mg po, three times a week; vaseline
Guo (2016)	TGP + NB-UVB	NB-UVB	Capsule, 0.6 g po tid	Three times a week
Zhu (2016)	I1: TGP + urea ointment I2: TGP + urea ointment	Urea ointment	I1: capsule, 0.6 g po tid I2: capsule, 0.6 g po bid	Topical use, bid
Huang (2015)	TGP + methotrexate tablet	Methotrexate tablet	Capsule, 0.4–0.6 g/d	7.5 mg po, once a week
Zou et al. (2015)	TGP + NB-UVB	NB-UVB	Capsule, 0.6 g po tid	Three times a week
Zhang et al. (2015)	TGP + NB-UVB	NB-UVB	Capsule, 0.6 g po tid	Once every 3 days
Cai et al. (2014)	TGP + calcipotriol ointment	0.005% calcipotriol Ointment	Capsule, 0.6 g po tid	Topical use, bid
Chen et al. (2014)	TGP + NB-UVB + AC	NB-UVB and acitretin capsule	Capsule, 0.6 g po tid	NB-UVB, three times a week; acitretin capsule, 0.5 mg/(kg·d)
Jiang et al. (2014)	TGP + AC + desonide cream	Acitretin capsule and desonide cream	Capsule, 0.6 g po tid	Acitretin capsule, 30–60 mg/d as initial oral dosage and adjusted dosage based on condition severity and medication tolerance and 10–30 mg/d as maintenance dosage; desonide cream, topical use, bid
Liu et al. (2014)	TGP + tretinoin ointment + mometasone furoate cream	0.01% tretinoin ointment and mometasone furoate cream	Capsule, 0.6 g po tid	0.01% tretinoin ointment, topical use, qd; mometasone furoate cream, topical use, qd
He et al. (2014)	TGP + NB-UVB + calcipotriol cream	NB-UVB and calcipotriol cream	Capsule, 0.6 g po tid	NB-UVB, qod; calcipotriol cream, topical use, bid
Yang et al. (2013)	TGP + NB-UVB	NB-UVB	Capsule, 0.6 g po tid	Three times a week
Jiang et al. (2013)	TGP + NB-UVB	NB-UVB	Capsule, 0.6 g po tid	NS
Hu et al. (2012a)	TGP + NB-UVB	NB-UVB	Capsule, 0.6 g po tid	qod
Du et al. (2012)	TGP + AC + hydrocortisone butyrate cream	Acitretin, hydrocortisone butyrate cream, and skin care oil	Capsule, 0.6 g po tid	Acitretin capsule, 30–60 mg/d; hydrocortisone butyrate cream; skin care oil, topical use, bid
He et al. (2012)	TGP + AC	Acitretin capsule	Capsule, 0.6 g po tid	0.5 mg/(kg·d) po
Hua et al. (2012)	TGP + compound glycyrrhizin tablet	Compound glycyrrhizin tablet	Capsule, 0.6 g po bid	75 mg po tid
Hu et al. (2012b)	TGP + tripterygium tablet + hydrocortisone ointment	Tripterygium tablet and hydrocortisone ointment	Capsule, 0.6 g po tid	Tripterygium tablet, 20 mg po bid; hydrocortisone ointment
Zhang et al. (2011)	TGP + 5% pine tar ointment	5% pine tar ointment	Capsule, 0.6 g po tid	Topical use, bid
Pang (2011)	TGP + NB-UVB + compound flumetasone ointment	NB-UVB and compound flumetasone ointment	Capsule, 0.6 g po tid	NB-UVB, qod; compound flumetasone ointment, topical use bid
Huang and Huang (2011)	TGP + AC + non glucocorticoids	Acitretin capsule and compound flumetasone ointment	Capsule, 0.6 g po tid	Acitretin capsule, 10 mg po tid; non glucocorticoids
Liu and Wang (2011)	TGP + compound glycyrrhizin injection	Compound glycyrrhizin injection	Capsule, 0.6 g po tid	80 mg/d ivgtt

TGP, total glucosides of paeony; NB-UVB, narrow-band ultraviolet B radiation; NS, not stated; po, administered orally; qd, once daily; qod, once every 2 days; tid, bid, twice daily; three times daily. ivgtt, intravenously guttae.

### 3.3 Risk of bias assessment

**FIGURE 2:**
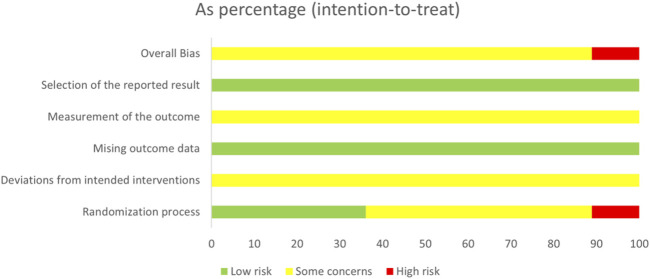
Risk of bias graph.

**FIGURE 3:**
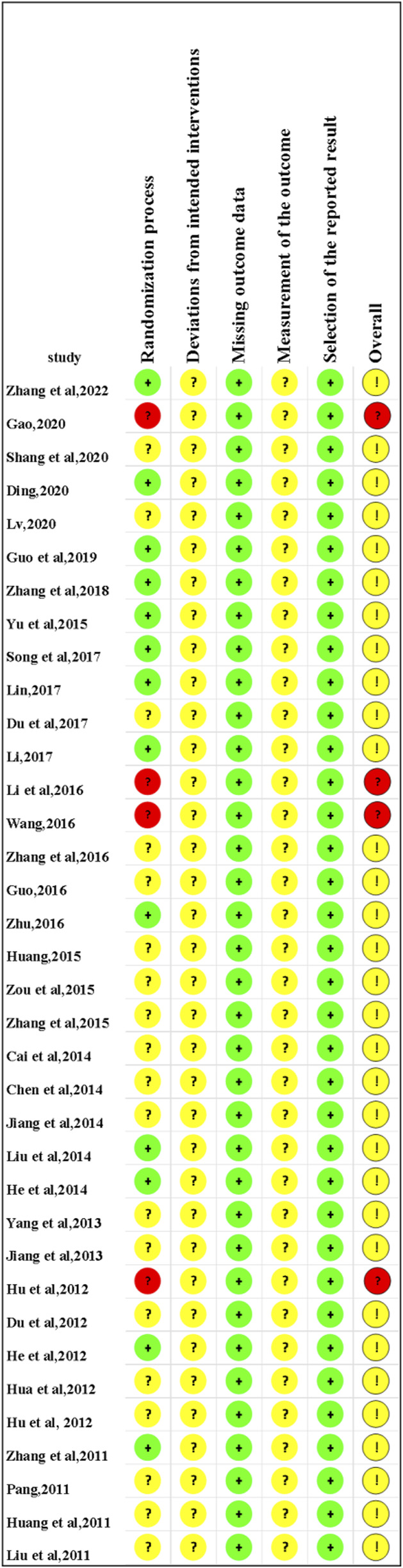
Risk of bias assessment.

In the domain of the randomization process, 13 studies (Yu et al., 2017; Ding, 2020; Guo and Xu, 2019; He et al., 2014; He et al., 2012; Li, 2017; Lin, 2017; Liu et al., 2014; Song et al., 2017; Zhang et al., 2022; Zhang et al., 2011; Zhu, 2016) were conducted using the random number tables for double-blind and were classified as low bias risk with the response of N/PN. Four studies (Gao, 2020; Hu et al., 2012a; Li et al., 2016; Wang, 2016) were rated as having a high bias risk with the response of Y/PY since randomization dispensing was based on the visiting order. In the domain of the missing outcome data and the selection of the reported result, all included studies were assessed as low risk of bias, with a response of Y/PY to outcome availability and data analysis consistency. Regarding the domain of deviations from intended interventions and outcome measurement, all included studies were considered to have some risk of bias because no detailed information on the abovementioned domains was detected. As for the domain of overall, 32 studies (Yu et al., 2017; Cai et al., 2014; Chen et al., 2014; Ding, 2020; Du et al., 2017; Du et al., 2012; Guo and Xu, 2019; Guo, 2016; He et al., 2014; He et al., 2012; Hu et al., 2012b; Hua et al., 2012; Huang, 2015; Huang and Huang, 2011; Jiang et al., 2014; Jiang et al., 2013; Li, 2017; Lin, 2017; Liu et al., 2014; Liu and Wang, 2011; Lv, 2020; Pang, 2011; Shang et al., 2020; Song et al., 2017; Yang et al., 2013; Zhang et al., 2015; Zhang et al., 2016; Zhang et al., 2022; Zhang and Wang, 2018; Zhang et al., 2011; Zhu, 2016; Zou et al., 2015) were ranked having the some concern, and 4 studies (Gao, 2020; Hu et al., 2012a; Li et al., 2016; Wang, 2016) were evaluated as having a high risk of bias, according to the results of the former five domains. The results of risk bias are shown in Figures 2, 3.

### 3.4 Meta-analysis of efficacy based on outcome measures

PASI was the main outcome measure used to assess psoriasis efficacy, with a score of 0–72 based on erythema, scaling, and induration (Griffiths et al., 2021). Subsequently, meta-analysis was conducted using specific outcome measures of PASI 75, PASI 60, and PASI 50, which were, respectively, defined as 75%, 60%, and 50% or greater reduction in the PASI score. Additionally, body surface area (BSA) and inflammatory factors were also evaluated.

#### 3.4.1 Based on PASI 75

**FIGURE 4:**
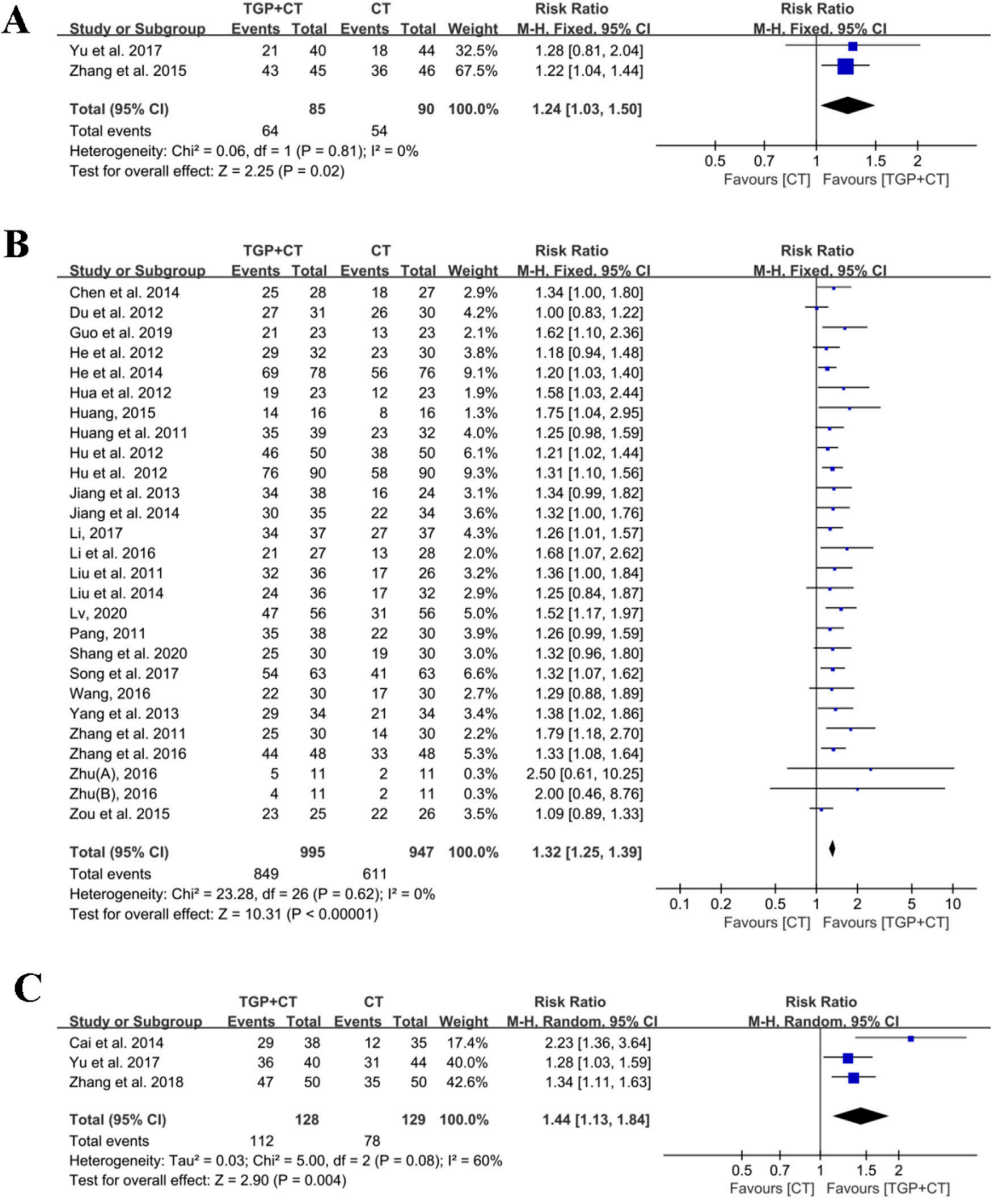
Meta-analysis based on outcome measures: **(A)** based on PASI 75; **(B)** based on PASI 60; and **(C)** based on PASI 50. TGP, total glucosides of paeony; CT, conventional therapy; PASI 75, 75% or greater reduction of the PASI score; PASI 60, 60% or greater reduction of the PASI score; PASI 50, and 50% or greater reduction of the PASI score; Zhu, 2016: 0.6 g TGP po tid; Zhu, 2016: 0.6 g TGP po bid.

Two studies (Yu et al., 2017; Zhang et al., 2015) estimated the clinical effect using PASI 75 as the outcome measure. With the acceptable heterogeneity detected (*I* ^*2*^ = 0% and *P* = 0.81), a fixed-effects model was selected for meta-analysis. The results showed there was a significant difference in TGP combined with conventional therapies when compared with conventional therapies used alone (RR = 1.24, 95% CI: 1.03 to 1.50, *P* = 0.02) (Figure 4A).

#### 3.4.2 Based on PASI 60

A total of 26 studies, involving 1,942 participants, utilized the outcome measures of PASI 60 for efficacy evaluation (Chen et al., 2014; Du et al., 2012; Guo and Xu, 2019; He et al., 2014; He et al., 2012; Hu et al., 2012a; Hu et al., 2012b; Hua et al., 2012; Huang, 2015; Huang and Huang, 2011; Jiang et al., 2014; Jiang et al., 2013; Li, 2017; Li et al., 2016; Liu et al., 2014; Liu and Wang, 2011; Lv, 2020; Pang, 2011; Shang et al., 2020; Song et al., 2017; Wang, 2016; Yang et al., 2013; Zhang et al., 2016; Zhang et al., 2011; Zhu, 2016; Zou et al., 2015). No significant heterogeneity was observed among the included studies (*I* ^*2*^ = 0% and *P* = 0.62), and thus, meta-analysis was implemented using a fixed-effects model. The findings indicated that TGP in combination with conventional therapies was superior to conventional therapies used alone in treating psoriasis (RR = 1.32, 95% CI: 1.25 to 1.39, *P* < 0.00001) (Figure 4B). The symmetry of the funnel plot and the detected result of the Egger’s test (*P* = 0.218 > 0.05) revealed no publication bias (Supplementary Figure S1A). Sensitivity analysis revealed the robustness and reliability of conclusion (Supplementary Figure S1B). Additionally, the results of meta-regression found no source of heterogeneity (Supplementary Figure S1C). Subgroup analysis was performed as follows:

##### 3.4.2.1 TGP plus monotherapy versus monotherapy

**FIGURE 5:**
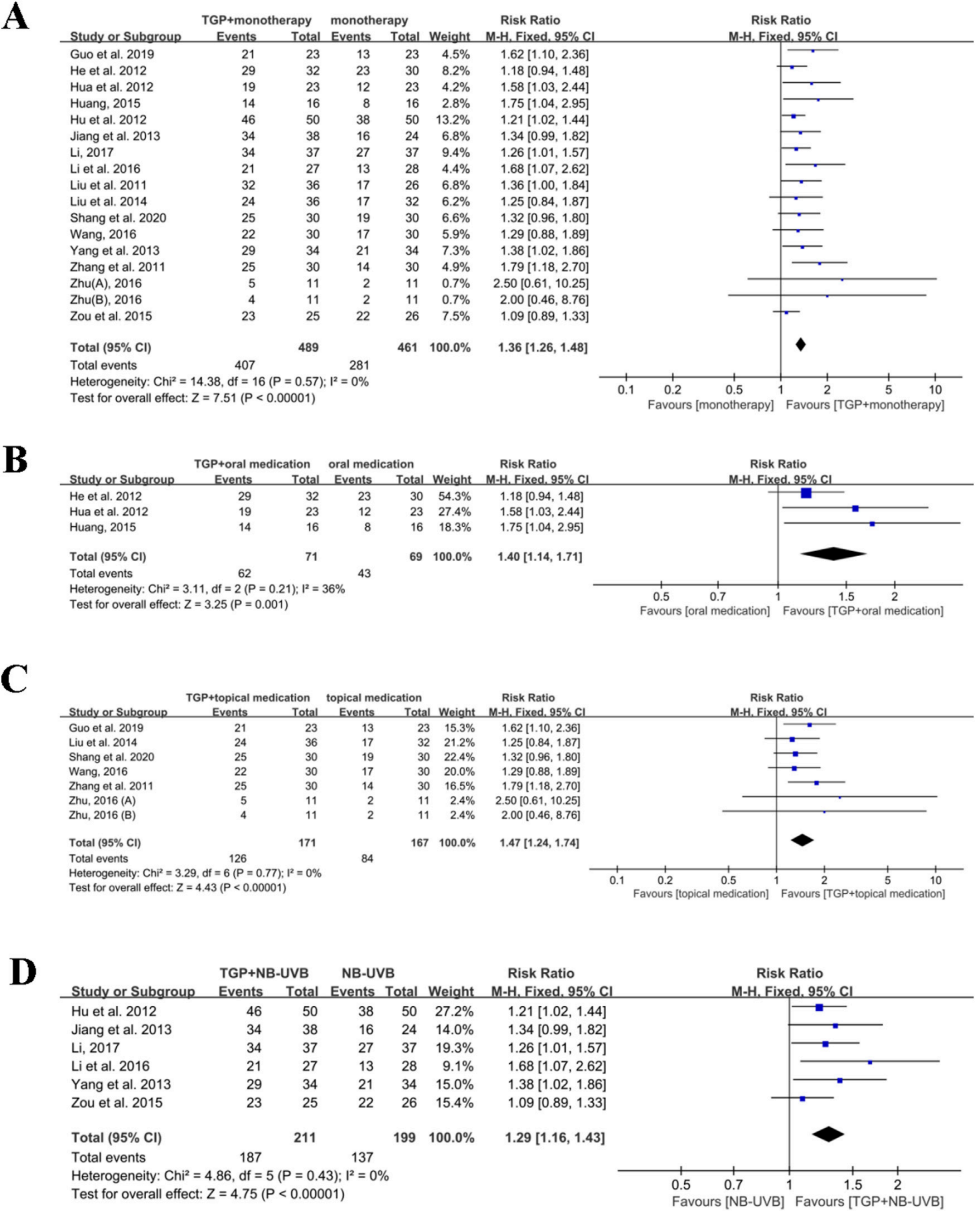
Meta-analysis of TGP plus monotherapy versus monotherapy. **(A)** TGP plus monotherapy versus monotherapy. **(B)** TGP plus oral medication versus oral medication. **(C)** TGP plus topical medication versus topical medication. **(D)** TGP plus NB-UVB versus NB-UVB. TGP, total glucosides of paeony; NB-UVB, narrow-band UVB; Zhu, 2016: 0.6 g TGP po tid; Zhu, 2016: 0.6 g TGP po bid.

A total of 16 studies with 950 participants reported the effect of TGP associated with monotherapy (Guo and Xu, 2019; He et al., 2012; Hu et al., 2012a; Hua et al., 2012; Huang, 2015; Jiang et al., 2013; Li, 2017; Li et al., 2016; Liu et al., 2014; Liu and Wang, 2011; Shang et al., 2020; Wang, 2016; Yang et al., 2013; Zhang et al., 2011; Zhu, 2016; Zou et al., 2015). Owing to no heterogeneity detected (*I* ^*2*^ = 0%, *P* = 0.57), a fixed-effects model was utilized for meta-analysis. The results demonstrated that TGP plus monotherapy was superior to monotherapy used alone (RR = 1.36, 95% CI: 1.26 to 1.48, *P* < 0.00001) (Figure 5A).

Three studies, containing 105 participants, mentioned the effects of TGP combined with oral medication (such as acitretin capsules) for psoriasis (He et al., 2012; Hua et al., 2012; Huang, 2015). A fixed-effects model was used for meta-analysis since acceptable heterogeneity was detected among the included studies (*I* ^*2*^ = 36%, *P* = 0.21). The results demonstrated that TGP in combination with oral medication had an advantage over oral medication alone (RR = 1.40, 95% CI: 1.14 to 1.71, *P* = 0.001) (Figure 5B).

Six studies with 338 participants evaluated the effects of TGP in conjunction with topical medication (such as tacrolimus ointment) (Guo and Xu, 2019; Liu et al., 2014; Shang et al., 2020; Wang, 2016; Zhang et al., 2011; Zhu, 2016). Due to no obvious heterogeneity observed (*I* ^*2*^ = 0%, *P* = 0.77), meta-analysis was performed using a fixed-effects model. The findings indicated that the combination of TGP and topical medication was more effective than topical medication alone (RR = 1.47, 95% CI: 1.24 to 1.74, *P* < 0.00001) (Figure 5C).

Six studies involving 410 participants indicated the effects of TGP with NB-UVB (Hu et al., 2012a; Jiang et al., 2013; Li, 2017; Li et al., 2016; Yang et al., 2013; Zou et al., 2015). Since no significant heterogeneity was found among the included studies (*I* ^*2*^ = 0% and *P* = 0.43), meta-analysis was accomplished with a fixed-effects model. The results showed that there was a statistically significant difference when comparing TGP plus NB-UVB with NB-UVB alone (RR = 1.29, 95% CI: 1.16 to 1.43, *P* < 0.00001) (Figure 5D).

##### 3.4.2.2 TGP plus multiple therapies versus multiple therapies alone

**FIGURE 6:**
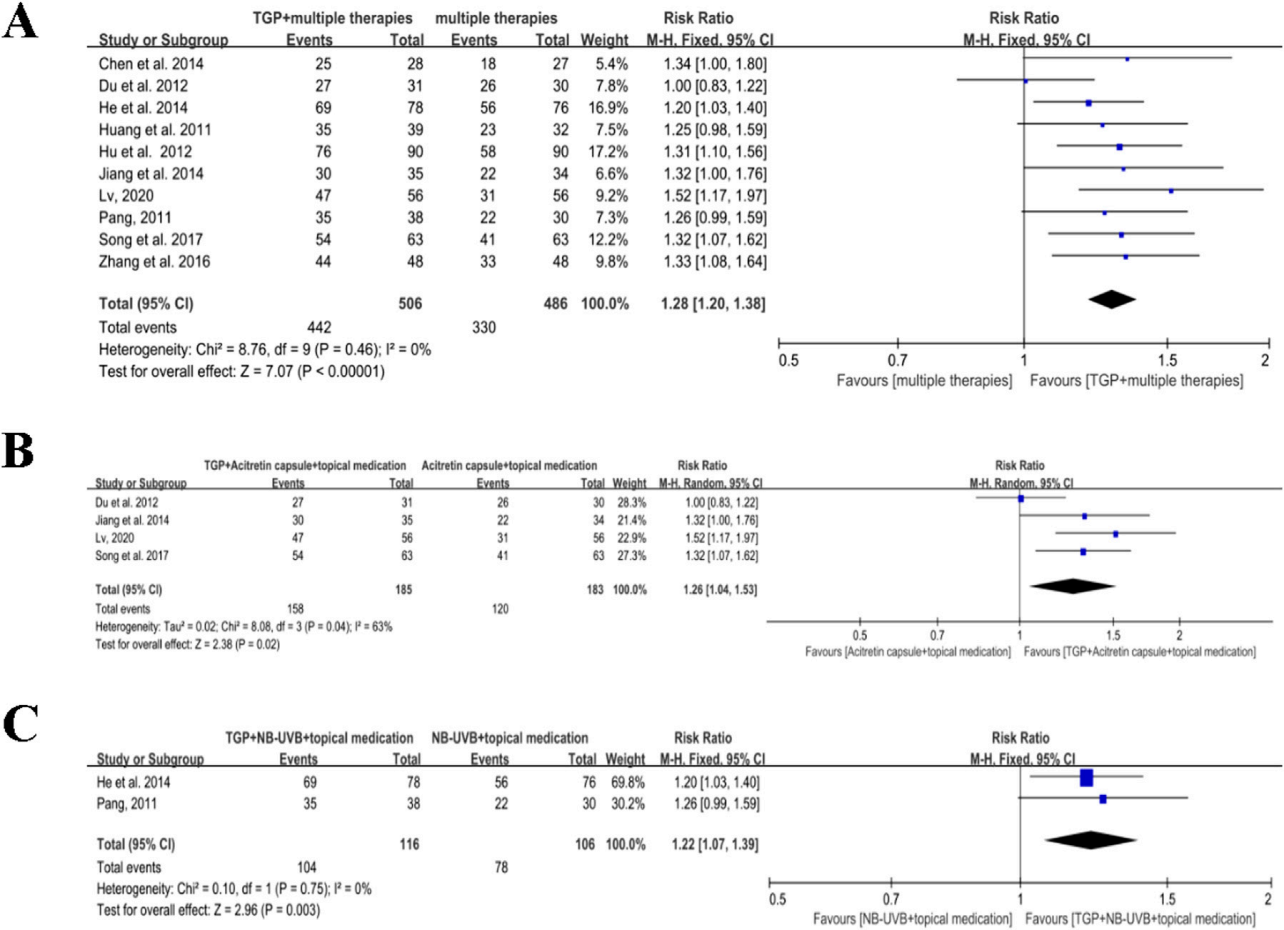
Meta-analysis of TGP plus multiple therapies versus multiple therapies. **(A)** TGP plus multiple therapies versus multiple therapies. **(B)** TGP plus the acitretin capsule and topical medication versus acitretin capsule and topical medication. **(C)** TGP plus NB-UVB and topical medication versus NB-UVB and topical medication. TGP, total glucosides of paeony; NB-UVB, narrow-band UVB.

A total of 10 studies, with a total of 992 participants, stated the effects of TGP associated with multiple therapies for psoriasis (Chen et al., 2014; Du et al., 2012; He et al., 2014; Hu et al., 2012b; Huang and Huang, 2011; Jiang et al., 2014; Lv, 2020; Pang, 2011; Song et al., 2017; Zhang et al., 2016). Since there was no significant heterogeneity among the included studies (*I* ^*2*^ = 0% and *P* = 0.46), meta-analysis was conducted using a fixed-effects model. The findings suggested that when combined with TGP, multiple therapies showed a more prominent effect than when used alone in the treatment of psoriasis (RR = 1.28, 95% CI: 1.20 to 1.38, *P* < 0.00001) (Figure 6A).

Four studies involving 368 participants reported the effects of TGP in combination with acitretin capsules and topical medication for psoriasis (Du et al., 2012; Jiang et al., 2014; Lv, 2020; Song et al., 2017). Due to substantial heterogeneity (*I* ^*2*^ = 63%, *P* = 0.04), a random effects model was applied for meta-analysis. The results revealed that when combined with TGP, multiple therapies of acitretin capsules and topical medication were not superior than using them alone (RR = 1.26, 95% CI: 1.04 to 1.53, and *P* = 0.02) (Figure 6B).

Two studies with 222 participants mentioned the effects of TGP in combination with NB-UVB and topical medication (He et al., 2014; Pang, 2011). Since no significant heterogeneity was detected (*I* ^*2*^ = 0% and *P* = 0.75), a fixed-effects model was used for meta-analysis. The findings showed that when combined with TGP, multiple therapies involving NB-UVB and topical medication were more effective than using them alone for psoriasis (RR = 1.22, 95% CI: 1.07 to 1.39, *P* = 0.003) (Figure 6C).

#### 3.4.3 Based on PASI 50

Three studies with 257 participants reported the effects of TGP and conventional therapies using the outcome measure of PASI 50 (Yu et al., 2017; Cai et al., 2014; Zhang and Wang, 2018). Given the significant heterogeneity (*I* ^*2*^ = 60%, *P* = 0.08), a random effects model was applied for meta-analysis. The findings revealed a statistical difference when TGP plus conventional therapies were compared with conventional therapies alone (RR = 1.44, 95% CI: 1.13 to 1.84, *P* = 0.004) (Figure 4C). Sensitivity analysis confirmed the robustness and reliability of the conclusion (Supplementary Figure S2).

#### 3.4.4 Based on BSA change

**FIGURE 7:**
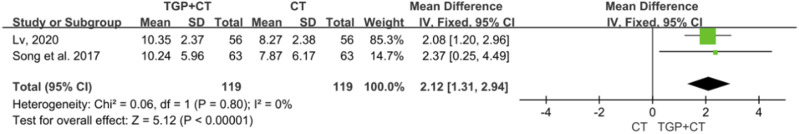
Meta-analysis based on BSA change. TGP, total glucosides of paeony; CT, conventional therapy; BSA, body surface area.

Two studies with 238 participants utilized the outcome measure of BSA to indicate the effects of TGP combined with conventional therapies (Lv, 2020; Song et al., 2017). Meta-analysis was conducted using a fixed-effects model, owing to no detected heterogeneity (*I* ^*2*^ = 0% and *P* = 0.80). The results illustrated that combining TGP with conventional therapies was superior to conventional therapies used alone (MD = 2.12, 95% CI: 1.31 to 2.94, *P* < 0.00001) (Figure 7).

#### 3.4.5 Based on inflammatory factor change

**FIGURE 8:**
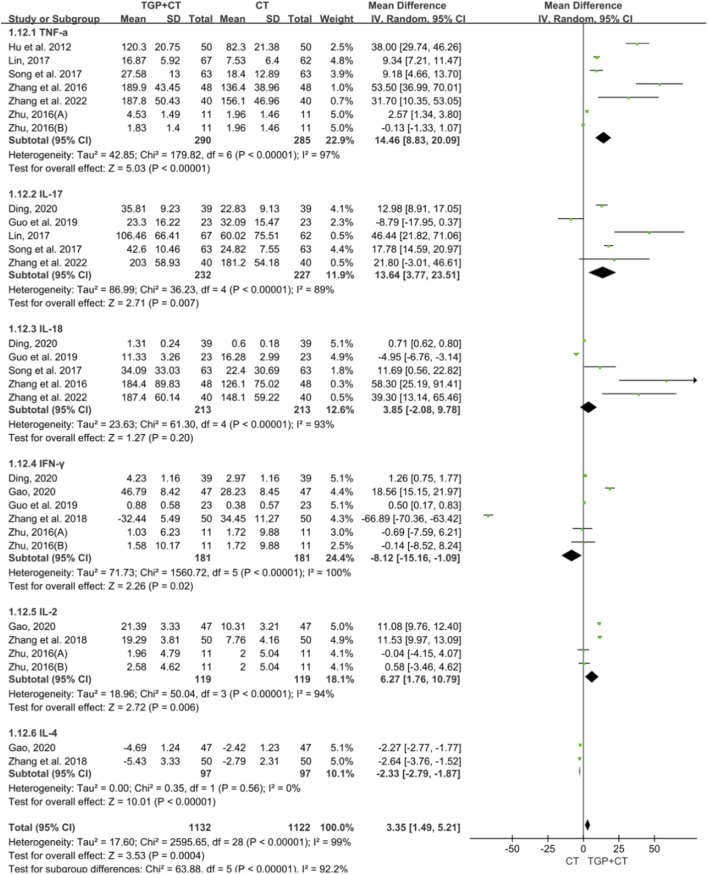
Meta-analysis based on inflammatory factor change. TGP, total glucosides of paeony; CT, conventional therapy; TNF-a, tumor necrosis factor-a; IL-17, interleukin-17; IL-18, interleukin-18; INF- Y interferon-y; IL-2, interleukin-2; IL-4, interleukin-4.

There were 10 studies with 882 participants mentioning the outcome measure of inflammatory factors. For significant heterogeneity, a random effects model was used for meta-analysis. The findings showed that when compared with conventional therapies used alone, TGP combined with conventional therapies increased the change in TNF-α (MD = 14.46, 95% CI: 8.83 to 20.09, *P* < 0.00001), IL-17 (MD = 13.64, 95% CI: 3.77 to 23.51, *P* = 0.007), IL-18 (MD = 3.85, 95% CI: -2.08 to 9.78, *P* = 0.20), and IL-2 (MD = 6.27, 95% CI: 1.76 to 10.79, *P* = 0.006) while decreasing in the change in INF-γ (MD = −8.12, 95% CI: -15.16 to −1.09, and *P* = 0.02) and IL-4 (MD = −2.33, 95% CI: -2.79 to −1.87, and *P* < 0.00001) (Figure 8). The symmetry observed in the funnel plot, combined with the result of the Egger’s test (*P* = 0.097 > 0.05), indicated the absence of publication bias (Supplementary Figure S3A). Sensitivity analysis confirmed the robustness and reliability of the conclusion (Supplementary Figure S3B), and the results of meta-regression revealed no source of heterogeneity (Supplementary Figure S3C).

### 3.5 Meta-analysis of efficacy based on the treatment period

Meta-analysis was conducted based on the treatment durations of 6, 8, and 12 weeks using the PASI 60 outcome measures.

#### 3.5.1 Treatment duration of 6 weeks

**FIGURE 9:**
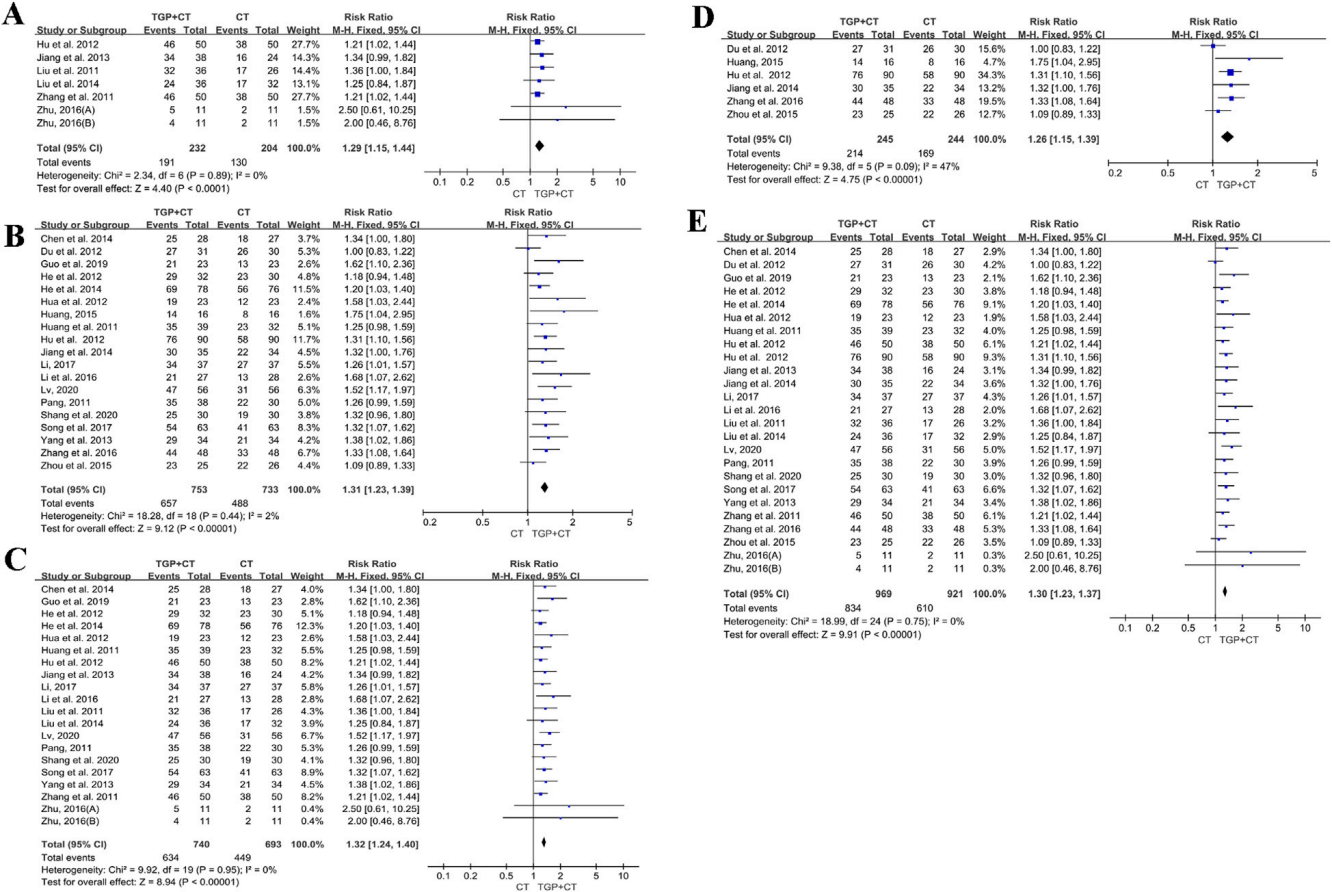
Meta-analysis based on treatment duration: **(A)** 6 weeks or less; **(B)** more than 6 weeks; **(C)** 8 weeks or less; **(D)** more than 8 weeks; and **(E)** 12 weeks or less. TGP, total glucosides of paeony; CT, conventional therapy.

For a duration of 6 weeks or less, six studies with 436 participants reported the effects of TGP with conventional therapies (Hu et al., 2012a; Jiang et al., 2013; Liu et al., 2014; Liu and Wang, 2011; Zhu, 2016). A fixed-effects model was used for meta-analysis, owing to low heterogeneity (*I* ^*2*^ = 0% and *P* = 0.89). The results indicated that TGP in combination with conventional therapies was superior to conventional therapies used alone (RR = 1.29, 95% CI: 1.15 to 1.44, and *P* < 0.00001) (Figure 9A). Sensitivity analysis showed the robustness and reliability of the conclusion (Supplementary Figure S4A).

For a duration of more than 6 weeks, 19 studies with 1,145 participants were enrolled to assess the efficacy of TGP with conventional therapies (Chen et al., 2014; Du et al., 2012; Guo and Xu, 2019; He et al., 2014; He et al., 2012; Hu et al., 2012b; Hua et al., 2012; Huang, 2015; Huang and Huang, 2011; Jiang et al., 2014; Li, 2017; Li et al., 2016; Lv, 2020; Pang, 2011; Shang et al., 2020; Song et al., 2017; Yang et al., 2013; Zhang et al., 2016; Zou et al., 2015). Meta-analysis was conducted using a fixed-effects model due to low heterogeneity (*I* ^*2*^ = 2%, *P* = 0.44). The findings revealed that TGP in combination with conventional therapies was superior to conventional therapies alone (RR = 1.31, 95% CI: 1.23 to 1.39, *P* < 0.00001) (Figure 9B). No publication bias was detected for the symmetry of the funnel plot and the detected result of Egger’s test (*P* = 0.428 > 0.05) (Supplementary Figure S4B). Sensitivity analysis manifested the reliability of the conclusion (Supplementary Figures S4B, C), and the findings of meta-regression found no source of heterogeneity (Supplementary Figure S4D).

#### 3.5.2 Treatment duration of 8 weeks

For a duration of 8 weeks or less, 19 studies with 1,433 participants reported the effects of TGP with conventional therapies (Chen et al., 2014; Guo and Xu, 2019; He et al., 2014; He et al., 2012; Hu et al., 2012a; Hua et al., 2012; Huang and Huang, 2011; Jiang et al., 2013; Li, 2017; Li et al., 2016; Liu et al., 2014; Liu and Wang, 2011; Lv, 2020; Pang, 2011; Shang et al., 2020; Song et al., 2017; Yang et al., 2013; Zhang et al., 2011; Zhu, 2016). Due to the observed heterogeneity, a fixed-effects model was used (*I* ^*2*^ = 0%, *P* = 0.95). The findings showed a significant difference, favoring the group of TGP combined with conventional therapies (RR = 1.32, 95% CI: 1.24 to 1.40, *P* < 0.00001) (Figure 9C). No publication bias was detected, owing to the symmetry of the funnel plot and the results of Egger’s test (*P* = 0.054 > 0.05) (Supplementary Figure S5A). Sensitivity analysis revealed the stability of the conclusion (Supplementary Figure S5B), and the results of meta-regression indicated no source of heterogeneity (Supplementary Figure S5C).

For a duration of more than 8 weeks, six studies containing 489 participants were analyzed (Du et al., 2012; Hu et al., 2012b; Huang, 2015; Jiang et al., 2014; Zhang et al., 2016; Zou et al., 2015). A fixed-effect model was utilized for the detected heterogeneity (*I* ^*2*^ = 47%, *P* = 0.09). The results indicated TGP with conventional therapies was superior when compared with conventional therapies alone (RR = 1.26, 95% CI: 1.15 to 1.39, *P* < 0.00001) (Figure 9D). Sensitivity analysis indicated the robustness and reliability of the conclusion (Supplementary Figure S5D).

#### 3.5.3 Treatment duration of 12 weeks

For a duration of 12 weeks or less, 24 studies with a total of 1,579 participants were evaluated (Chen et al., 2014; Du et al., 2012; Guo and Xu, 2019; He et al., 2014; He et al., 2012; Hu et al., 2012a; Hu et al., 2012b; Hua et al., 2012; Huang and Huang, 2011; Jiang et al., 2013; Jiang et al., 2014; Li, 2017; Li et al., 2016; Liu et al., 2014; Liu and Wang, 2011; Lv, 2020; Pang, 2011; Shang et al., 2020; Song et al., 2017; Yang et al., 2013; Zhang et al., 2011; Zhang et al., 2016; Zou et al., 2015; Zhu, 2016). A fixed-effects model was selected for the low heterogeneity (*I* ^*2*^ = 0% and *P* = 0.75). The results reported the superior effect of TGP combined with conventional therapies (RR = 1.30, 95% CI: 1.23 to 1.37, *P* < 0.00001) (Figure 9E). With the symmetry of the funnel plot and the findings of Egger’s test (*P* = 0.425 > 0.05), there was no publication bias (Supplementary Figure S6A). Sensitivity analysis proved the stability of the conclusion (Supplementary Figure S6B). Additionally, the results of meta-regression showed no source of heterogeneity (Supplementary Figure S6C). There was only one study (Huang, 2015) with a duration of more than 12 weeks.

### 3.6 Meta-analysis of safety

Meta-analysis of adverse events was evaluated on the basis of laboratory examination and clinical symptoms.

#### 3.6.1 Adverse events based on laboratory examination

**FIGURE 10:**
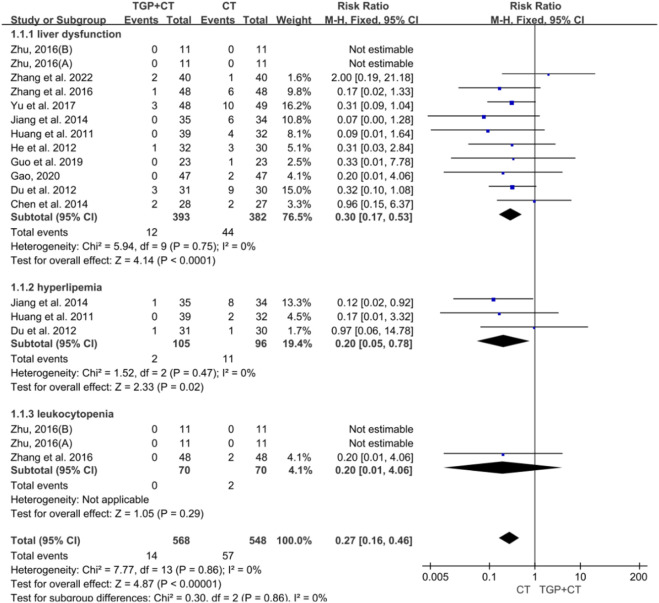
Meta-analysis of adverse events based on laboratory examination. TGP, total glucosides of paeony; CT, conventional therapy.

A total of 11 studies with 775 participants reported the occurrence of liver dysfunction (Yu et al., 2017; Chen et al., 2014; Du et al., 2012; Gao, 2020; Guo and Xu, 2019; He et al., 2012; Huang and Huang, 2011; Jiang et al., 2014; Zhang et al., 2016; Zhang et al., 2022; Zhu, 2016). Three studies with 201 participants reported the adverse event of leukocytopenia (Du et al., 2012; Huang and Huang, 2011; Jiang et al., 2014), while two studies containing 129 participants reported hyperlipidemia (Zhang et al., 2016; Zhu, 2016). Given low heterogeneity, a fixed-effects model was selected for meta-analysis. The findings revealed significant differences, favoring the conventional therapy group in liver dysfunction (RR = 0.03, 95% CI: 0.17 to 0.53, *P* < 0.00001), hyperlipidemia (RR = 0.20, 95% CI: 0.05 to 0.78, *P* = 0.02), and leukocytopenia (RR = 0.20, 95% CI: 0.01 to 4.06, *P* = 0.29) (Figure 10). Concerning liver dysfunction, no publication bias was found according to the symmetry of the funnel plot and the detected result of Egger’s test (*P* = 0.766 > 0.05) (Supplementary Figure S7A). Sensitivity analysis manifested the robustness of the conclusion (Supplementary Figure S7B), and no source of heterogeneity was observed by meta-regression (Supplementary Figure S7C).

#### 3.6.2 Adverse events based on clinical symptoms

**FIGURE 11:**
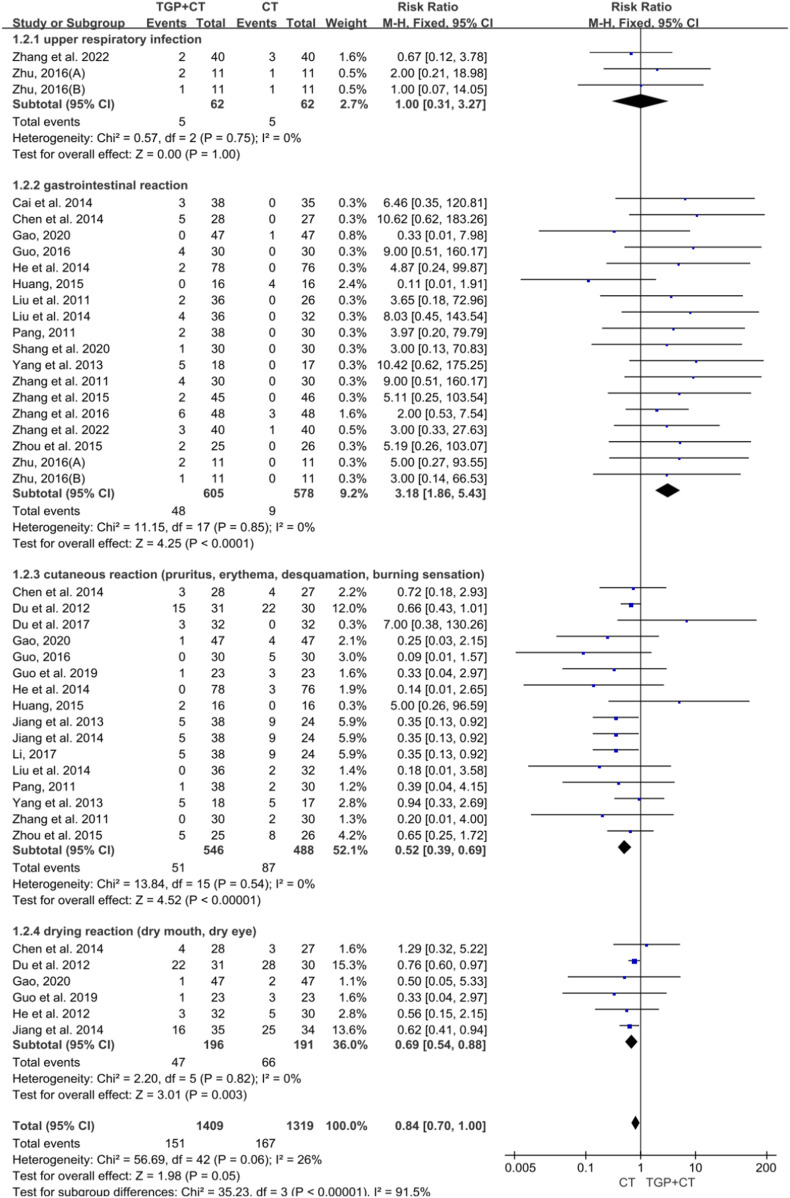
Meta-analysis of adverse events based on clinical symptoms. TGP, total glucosides of paeony; CT, conventional therapy.

Two studies with 113 participants reported the occurrence of upper respiratory infection (Zhang et al., 2022; Zhu, 2016), while 18 studies involving 1,352 participants mentioned the adverse event of gastrointestinal reactions (Cai et al., 2014; Chen et al., 2014; Gao, 2020; Guo, 2016; He et al., 2014; Huang, 2015; Hu et al., 2012b; Liu et al., 2014; Liu and Wang, 2011; Pang, 2011; Shang et al., 2020; Yang et al., 2013; Zhang et al., 2015; Zhang et al., 2016; Zhang et al., 2022; Zhang et al., 2011; Zhu, 2016; Zou et al., 2015). There were 16 studies with 1,041 participants that mentioned cutaneous reactions (Du et al., 2017; Du et al., 2012; Gao, 2020; Guo and Xu, 2019; Guo, 2016; He et al., 2014; Huang, 2015; Jiang et al., 2014; Jiang et al., 2013; Li, 2017; Liu et al., 2014; Pang, 2011; Yang et al., 2013; Zhang et al., 2011; Zou et al., 2015), while six studies with 387 participants mentioned drying reactions (Chen et al., 2014; Du et al., 2012; Gao, 2020; Guo and Xu, 2019; He et al., 2012; Jiang et al., 2014). For no detected significant heterogeneity, a fixed-effects model was used for meta-analysis as follows: upper respiratory infection (RR = 1.00, 95% CI: 0.31 to 3.27, *P* = 1.00), gastrointestinal reaction (RR = 3.18, 95% CI: 1.86 to 5.43, *P* < 0.00001), cutaneous reaction (RR = 0.52, 95% CI: 0.39 to 0.69, *P* < 0.00001), and drying reaction (RR = 0.69, 95% CI: 0.54 to 0.88, *P* = 0.003) (Figure 11). Concerning gastrointestinal reactions, no obvious publication bias was detected for the symmetrical funnel plot and the detected result of Egger’s test (*P* = 0.440 > 0.05) (Supplementary Figure S8A). Sensitivity analysis indicated the reliability of the conclusion (Supplementary Figure S8B), and the findings of meta-regression showed no source of heterogeneity (Supplementary Figure S8C). Concerning cutaneous reactions, the results of the symmetrical funnel plot and Egger’s test (*P* = 0.613 > 0.05) indicated no obvious publication bias (Supplementary Figure S8D). Sensitivity analysis confirmed the stability of above findings (Supplementary Figure S8E). Moreover, no source of heterogeneity was indicated by meta-regression (Supplementary Figure S8F).

## 4 Discussions

### 4.1 Summary of the findings

The aim of this systematic review and meta-analysis was to critically elucidate the add-on effects of TGP on conventional therapies in the treatment of psoriasis. A total of 328 relevant studies were retrieved from seven databases, and 36 RCTs with 3,140 participants were carefully included. In terms of efficacy, the findings indicated that TGP combined with conventional therapies superior to conventional therapies used alone, with outcome measures of PASI 75 (RR = 1.24, 95% CI: 1.03 to 1.50, *P* = 0.02), PASI 60 (RR = 1.32, 95% CI: 1.25 to 1.39, *P* < 0.00001), and PASI 50 (RR = 1.44, 95% CI: 1.13 to 1.84, *P* = 0.004). Surprisingly, when compared with conventional therapies used alone, the combination of TGP with different types of conventional therapies, such as oral medication (RR = 1.40, 95% CI: 1.14 to 1.71, *P* = 0.001), topical medication (RR = 1.47, 95% CI: 1.24 to 1.74, *P* < 0.00001), and NB-UVB (RR = 1.29, 95% CI: 1.16 to 1.43, *P* < 0.00001), showed higher efficacy. In terms of BSA, combining TGP with conventional therapies was superior to conventional therapies used alone (RR = 2.12, 95% CI: 1.31 to 2.94, *P* < 0.00001). In terms of inflammatory factors, TGP combined with conventional therapies showed significant differences in TNF-α, IL-17, IL-18, IL-2, INF-γ, and IL-4 compared to conventional therapies used alone. In the terms of treatment duration, there were significant differences in the treatment of TGP combined with conventional therapies at 6, 8, and 12 weeks compared with conventional therapies used alone. These findings demonstrated that TGP shows potential to improve the efficacy of conventional medicines.

In terms of adverse events, a total of 31 studies mentioned the occurrence of adverse events when treated with TGP and conventional therapies for psoriasis. Concerning the adverse events of laboratory examination, differences were detected, favoring the conventional therapy group in liver dysfunction, hyperlipidemia, and leukocytopenia. Concerning adverse events of clinical symptoms, there were differences favoring TGP combined with conventional therapies in relieving cutaneous and drying reactions. Additionally, differences were detected favoring the conventional therapy group in the gastrointestinal reaction, and there was no difference observed in the upper respiratory infection.

### 4.2 Potential therapeutic mechanisms of TGP on conventional therapies

Similar to the findings of this study, experimental research studies indicated that TGP can eliminate the epidermal layer thickness and inhibit skin inflammation infiltration and angiogenesis in the imiquimod-induced psoriasis-like mouse models (Zhao et al., 2024). Published animal experiments showed that TGP could regulate skin inflammation in psoriasis-like mouse models and is induced by imiquimod by activating psoriatic dermal mesenchymal stem cells and modulating vascular endothelial growth factor (VEGF) expression (Lei et al., 2023; Zhang et al., 2021). Additionally, TGP could inhibit the overactivation of keratinocytes and the differentiation of T helper 17 (TH17) cells, markedly downregulating the gene expression of Th17-related cytokines (Zhang et al., 2023; Li et al., 2019). Paeoniflorin, the active ingredient of TGP, prominently restrained the proinflammatory cytokines and effector molecules such as macrophage inflammatory protein-2 (MIP-2), inducible nitric oxide synthase (iNOS), and TNF-α by macrophages and neutrophils (Zhang and Wei, 2019; Sun et al., 2015). Overall, the available evidence reveals that TGP effectively improves imiquimod-induced psoriatic skin lesions at immune cell, keratinocyte, and dermal mesenchymal stem cell levels by regulating immune imbalance, inducing inflammatory response, and modulating angiogenesis. However, as for the add-on effect of TGP on conventional therapies, there is still a lack of relevant experimental research on elucidating the underlying mechanisms of action. Previous clinical studies suggested that when compared with conventional therapies used alone, such as NB-UVB, cyclosporine, acitretin capsule, and tacrolimus ointment, the inclusion of TGP could reduce the proinflammatory factor levels of IL-17, IL-23, IL-4, and IFN-γ to improve the progression of psoriasis (Ren and Zhao, 2021; Yi, 2015; Lin et al., 2022; Ding, 2020; Griffiths et al., 2021).

Concerning safety, the combined application of TGP proved beneficial in reducing the adverse events related to acitretin, such as dry skin and liver dysfunction (Yu et al., 2017; Du et al., 2012; Huang and Huang, 2011; Zhang et al., 2016; Zou et al., 2015), owing to the immunoregulatory effect of TGP (Zhang and Wei, 2019). In addition, conventional therapy of NB-UVB might cause adverse events of cutaneous pruritus and erythema during the treatment of psoriasis (Du et al., 2017; Guo, 2016; Jiang et al., 2013), and the combined use of TGP, which has anti-inflammatory effect, may alleviate these conditions (Zhang and Wei, 2019). Noteworthily, gastrointestinal disorders such as diarrhea and abdominal discomfort were more likely to occur when TGP was combined, possibly due to the TGP administration at high dose (Zhu, 2016; Zhang et al., 2022; Pang, 2011). Thus, more attention should be paid to administering the application TGP, including dosage, frequency, and duration. In general, it was believed that TGP had a safety stable profile when combined with conventional therapies in treating psoriasis.

### 4.3 Strengths of this study compared with the previous study

According to the Cochrane Library’s guidelines (Karunananthan et al., 2020; Smith and Ho, 2023), systematic reviews and meta-analyses for a specific treatment should be updated periodically to provide clinicians with the most recent and higher-quality clinical evidence. Compared with the previously published article (Zheng et al., 2019), the strengths of this article lie in the following points: first, to assess efficacy, this study employs validated outcome measures recognized by international guidelines, such as PASI 75, PASI 50, and BSA (Elmets et al., 2021; Nast et al., 2018; Nast et al., 2020; Psoriasis Committee, Dermatology and Venereology Branch, Chinese Medical Association, 2023). Second, this study incorporated the evaluation of inflammatory cytokines from preclinical studies, which are closely related to the progression of psoriasis (Griffiths et al., 2021). Third, to assess safety, this study categorized adverse events into two distinct classes, namely, laboratory examination and clinical symptoms, which facilitated a comprehensive, multi-dimensional observation of adverse reactions, following the treatment of TGP with conventional therapies. Additionally, regarding risk bias assessment, this study employed the latest RoB 2.0 tool published by the Cochrane Library, providing a broader and more comprehensive scope to furnish supplementary details of bias risks derived from the included RCTs (Nejadghaderi et al., 2024; Sterne et al., 2019).

### 4.4 Limitations to this study

Although rigorous methodology was utilized for data analysis, there were still limitations that needed to be addressed. On one hand, due to incomplete raw data, the methodological quality of the majority of the included studies was generally poor, which may have an impact on the accurate interpretation of this systematic review and meta-analysis. On the other hand, included studies with small sample sizes and single research center somewhat limited the universality of the findings. In addition, there might be a language bias, owing to initial studies that only search English and Chinese databases.

### 4.5 Implications for future studies

Based on the abovementioned findings, strengths, and limitations of this study, suggestions for future clinical practice and experimental research are made. First of all, rigorously designed RCTs accompanied by multiple centers and enlarged sample sizes are urgently needed to promote the evidence in support of TGP combined with conventional therapies for psoriasis. Subsequently, the standard guidelines of Consolidated Standards of Reporting Trials (CONSORT) should be strictly adhered to throughout the process of RCTs, especially during the implementation of randomization, blinding, and allocation concealment. Moreover, in order to fully demonstrate the underlying mechanisms of action of TGP combined with conventional therapies for psoriasis, intensive experimental research from different molecular, cell, and tissue levels is required. This could provide a novel optimized therapeutic strategy for psoriasis and promote the evidence for the application of conventional therapies and TGP. Further explorations on optimal treatment administration and long-term safety profile assessment could potentially provide useful guidance for future studies.

## 5 Conclusion

This systematic review and meta-analysis demonstrated several therapeutic sources of evidence supporting the addition of TGP to conventional therapies in treating psoriasis. Additionally, when combined with TGP, conventional therapies such as acitretin capsule and NB-UVB have been shown to improve clinical efficacy and reduce the incidence of adverse events. However, the limited methodological quality and small sample sizes of included studies may impede the accurate interpretation of the findings. To better clarify the add-on effects of TGP on conventional therapies for psoriasis, comprehensively designed and large-scale research studies are further needed.

## Data Availability

The original contributions presented in the study are included in the article/Supplementary Material; further inquiries can be directed to the corresponding authors.
