# Supplementary material for: Add-on effects of total glucosides of paeony on conventional therapies for psoriasis: a systematic review and meta-analysis of randomized controlled trials
Source: Front Pharmacol. 2025 Feb 19;16:1527288. doi: 10.3389/fphar.2025.1527288 (PMC11880009; doi:10.3389/fphar.2025.1527288)
Supplement: Supplementary file 1 [file Table1.docx]

Supplementary Material

**Table of contents**

[1 Supplementary Figures 2](#_Toc3421)

[Supplementary Figure S1. Publication bias, sensitivity analysis and meta-regression based on PASI 60 2](#_Toc31272)

[Supplementary Figure S2. Sensitivity analysis based on PASI 50 3](#_Toc2737)

[Supplementary Figure S3. Publication bias, sensitivity analysis and meta-regression of based on inflammatory factors 4](#_Toc15112)

[Supplementary Figure S4. Publication bias, sensitivity analysis and meta-regression based on treatment duration of 6 weeks 5](#_Toc4124)

[Supplementary Figure S5. Publication bias, sensitivity analysis and meta-regression based on treatment duration of 8 weeks 6](#_Toc26765)

[Supplementary Figure S6. Publication bias, sensitivity analysis and meta-regression based on treatment duration of 12 weeks 7](#_Toc5019)

[Supplementary Figure S7. Publication bias, sensitivity analysis and meta-regression based on adverse events of laboratory examination 8](#_Toc5019)

[Supplementary Figure S8. Publication bias, sensitivity analysis and meta-regression based on adverse events of clinical symptoms 9](#_Toc5019)

[2 Supplementary Table 11](#_Toc31793)

[Supplementary Table 1. Characteristics of included studies 1](#_Toc2177)1

[Supplementary Table 2. Detailed information concerning the intervention/comparators of included studies 1](#_Toc2177)4

[3 Supplementary File 16](#_Toc31793)

[Supplementary File S1. The PRISMA checklist of this meta-analysis 1](#_Toc2177)6

# Supplementary Figures

# Supplementary Figure S1. Publication bias, sensitivity analysis and meta-regression based on PASI 60

(A) Funnel plots for publication bias. (B) Sensitivity analysis. (C) Meta-regression.


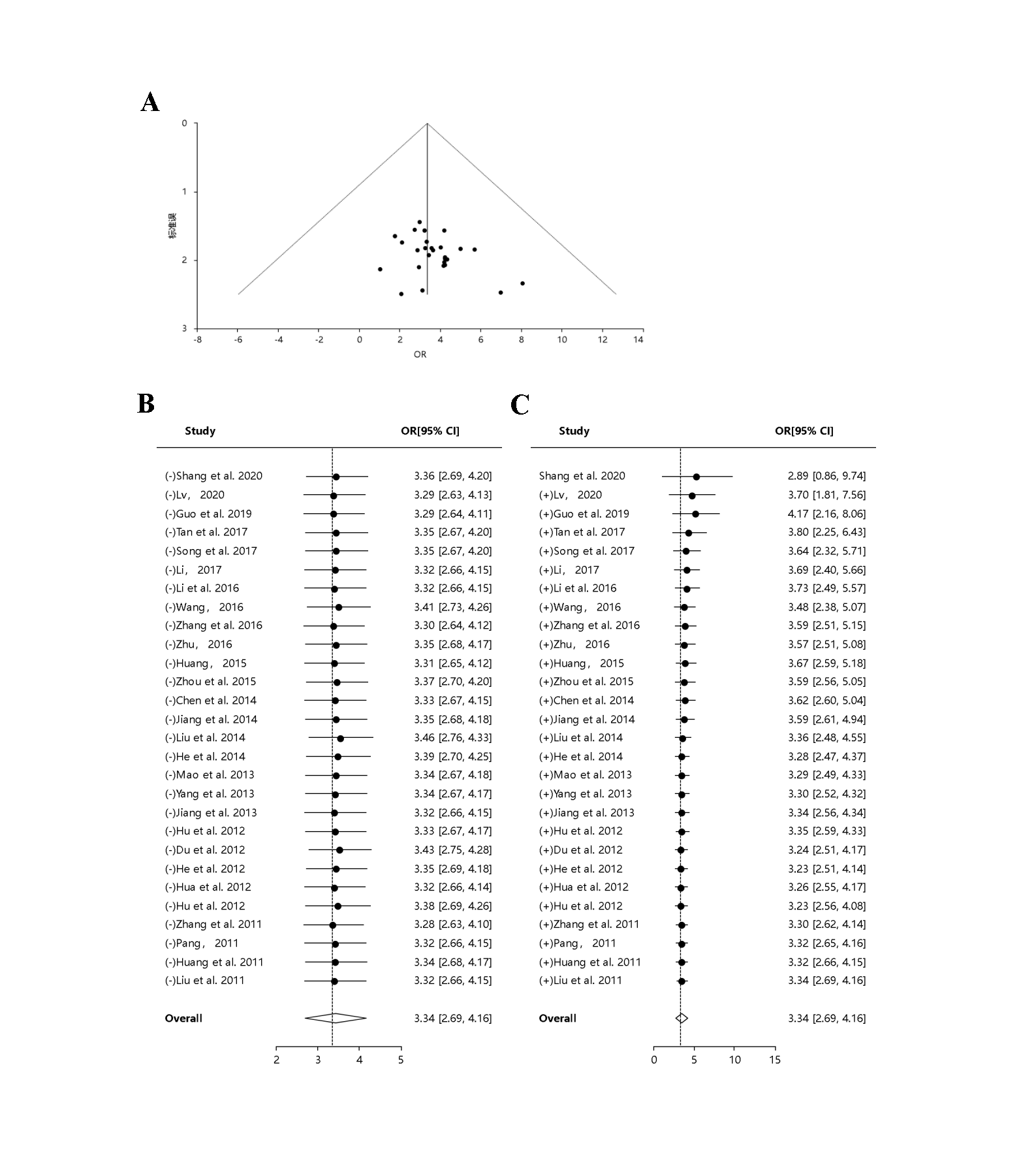


# Supplementary Figure S2. Sensitivity analysis based on PASI 50


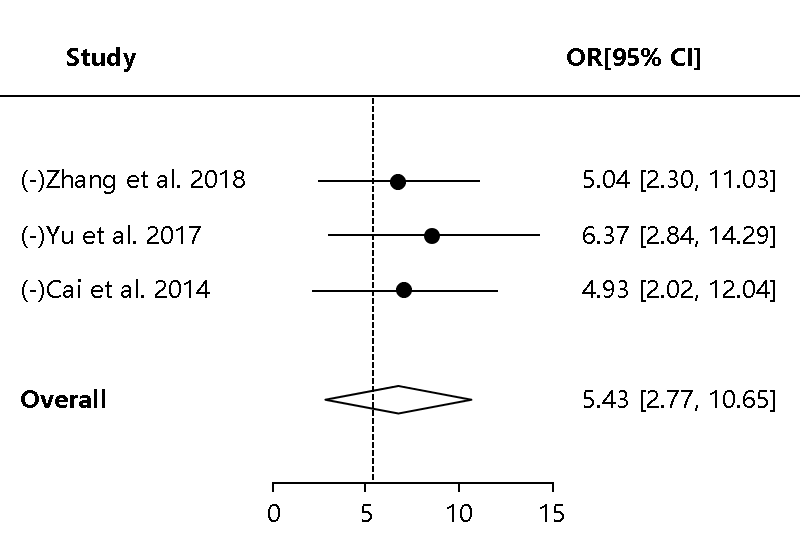


# Supplementary Figure S3. Publication bias, sensitivity analysis and meta-regression based on inflammatory factors

(A) Funnel plots for publication bias. (B) Sensitivity analysis. (C) Meta-regression.


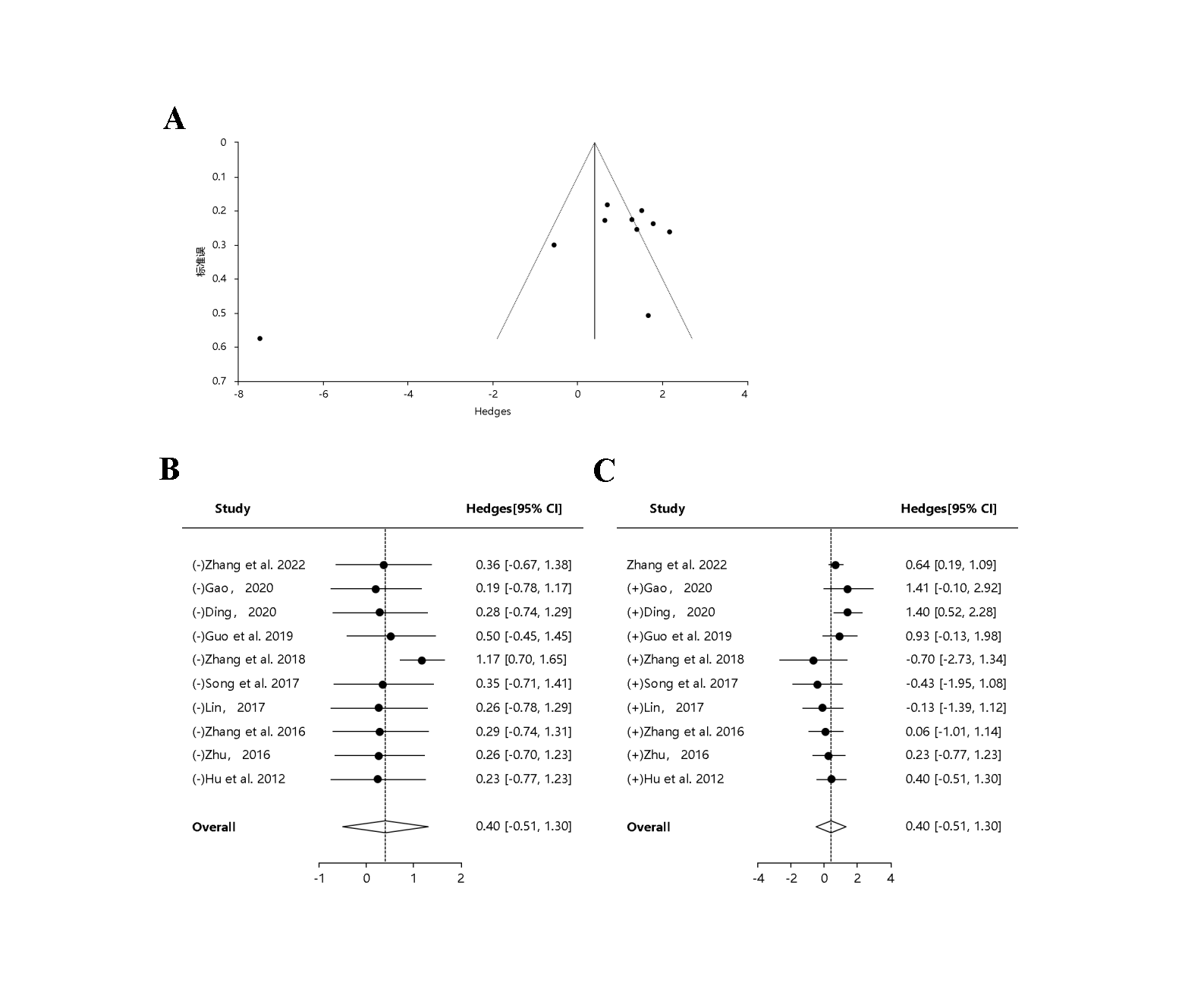


# Supplementary Figure S4. Publication bias, sensitivity analysis and meta-regression based on treatment duration of 6 weeks

1. Sensitivity analysis for duration of 6 weeks or less. (B) Funnel plots for publication bias for duration of longer than 6 weeks. (C) Sensitivity analysis for duration of longer than 6 weeks. (D) Meta-regression for duration of longer than 6 weeks.


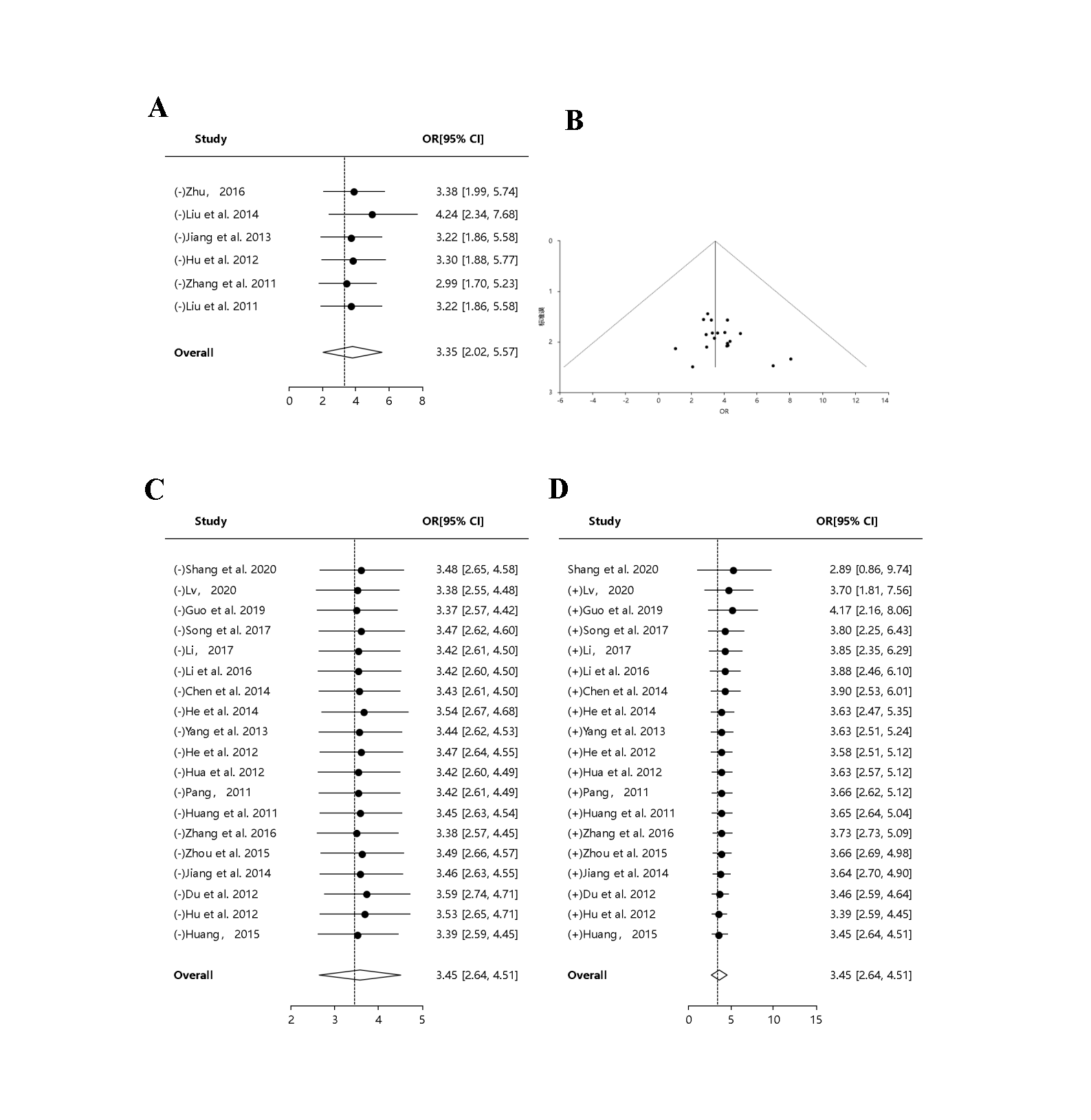


# Supplementary Figure S5. Publication bias, sensitivity analysis and meta-regression based on treatment duration of 8 weeks

(A) Funnel plots for publication bias for duration of 8 weeks or less. (B) Sensitivity analysis for duration of 8 weeks or less. (C) Meta-regression for duration of 8 weeks or less. (D) Sensitivity analysis for duration of longer than 8 weeks.


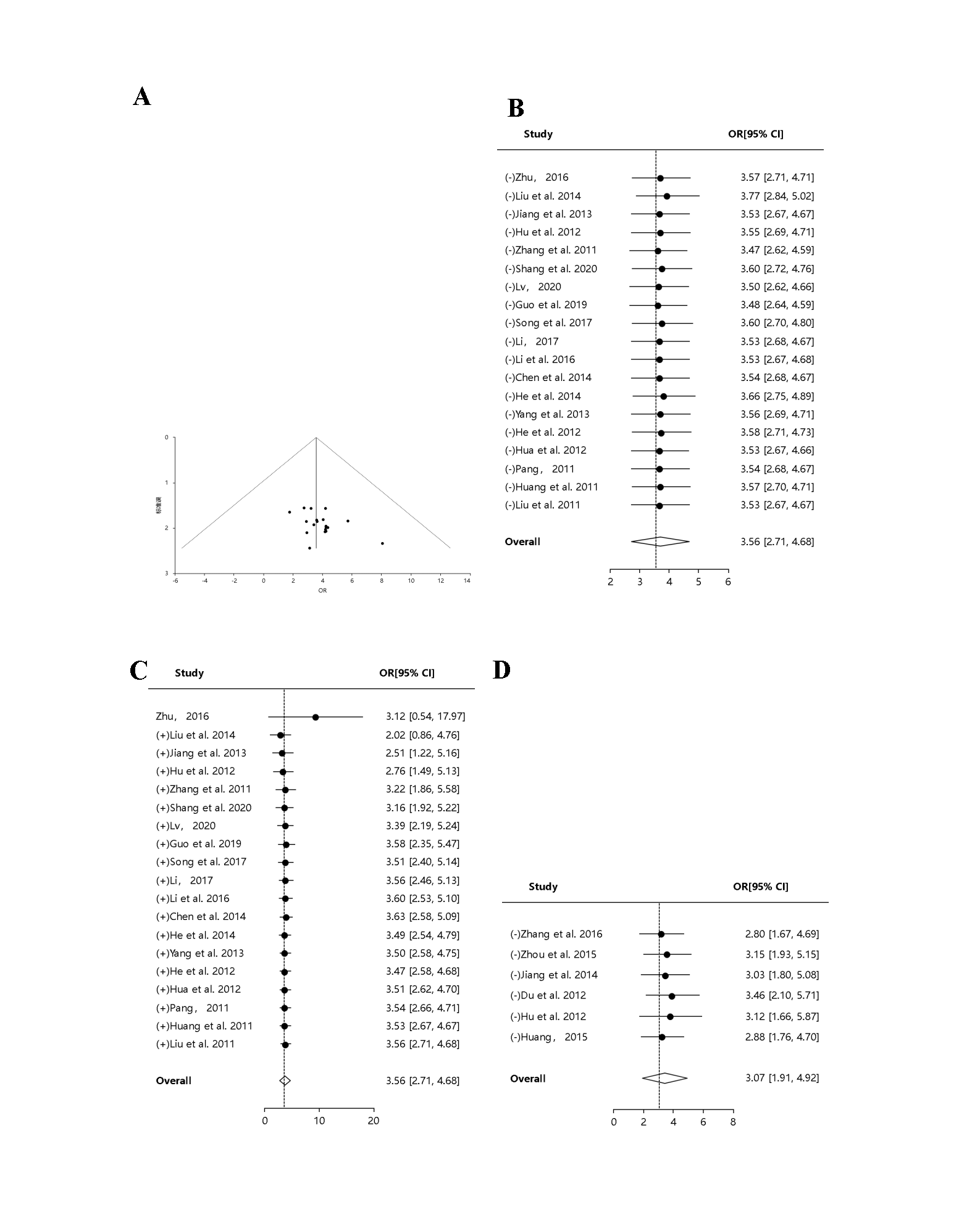


# Supplementary Figure S6. Publication bias, sensitivity analysis and meta-regression based on treatment duration of 12 weeks

(A) Funnel plots for publication bias for duration of 12 weeks or less. (B) Sensitivity analysis for duration of 12 weeks or less. (C) Meta-regression for duration of 12 weeks or less.


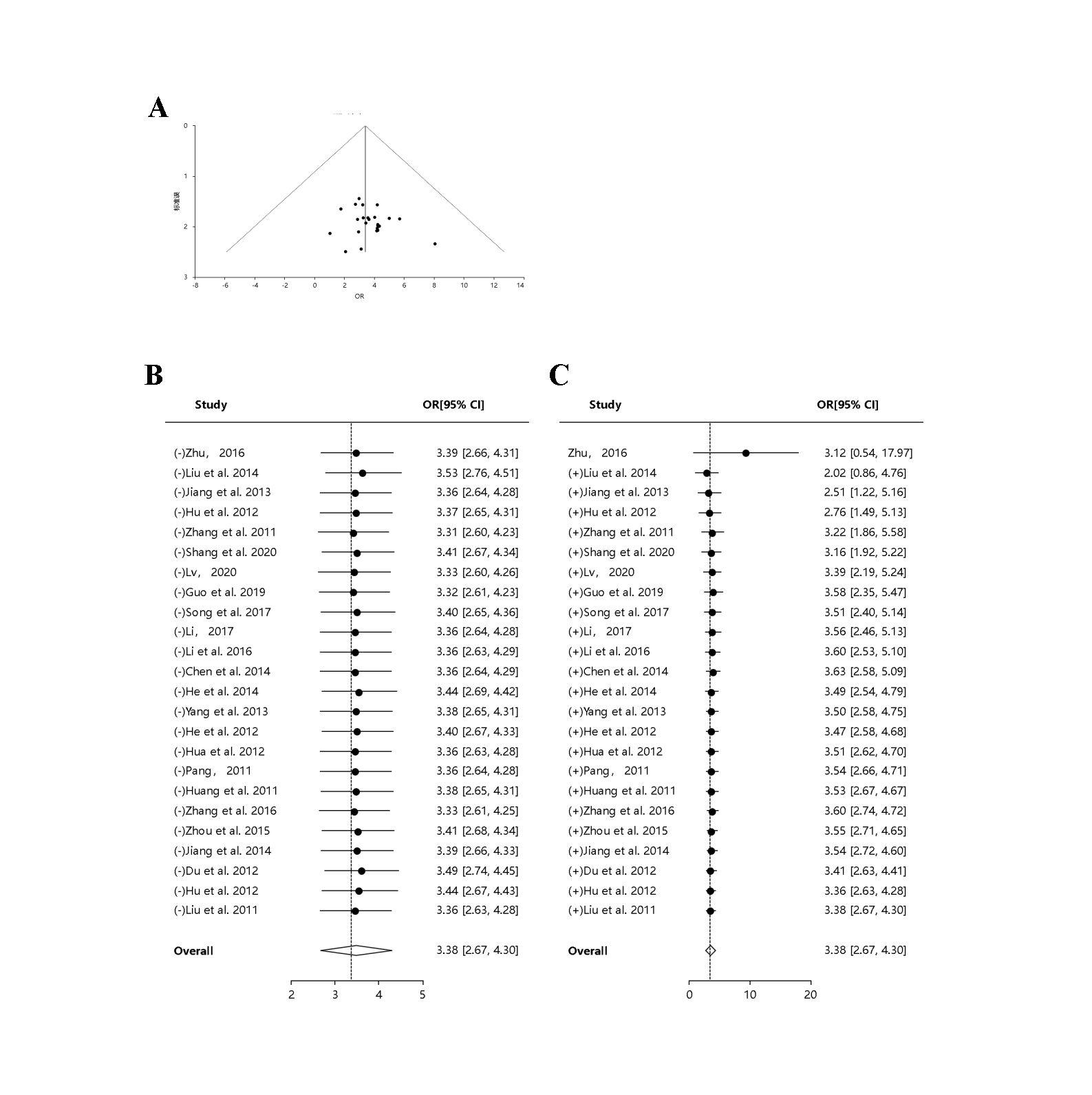


# Supplementary Figure S7. Publication bias, sensitivity analysis and meta-regression based on adverse events of laboratory examination

(A) Funnel plots for publication bias. (B) Sensitivity analysis. (C) Meta-regression.


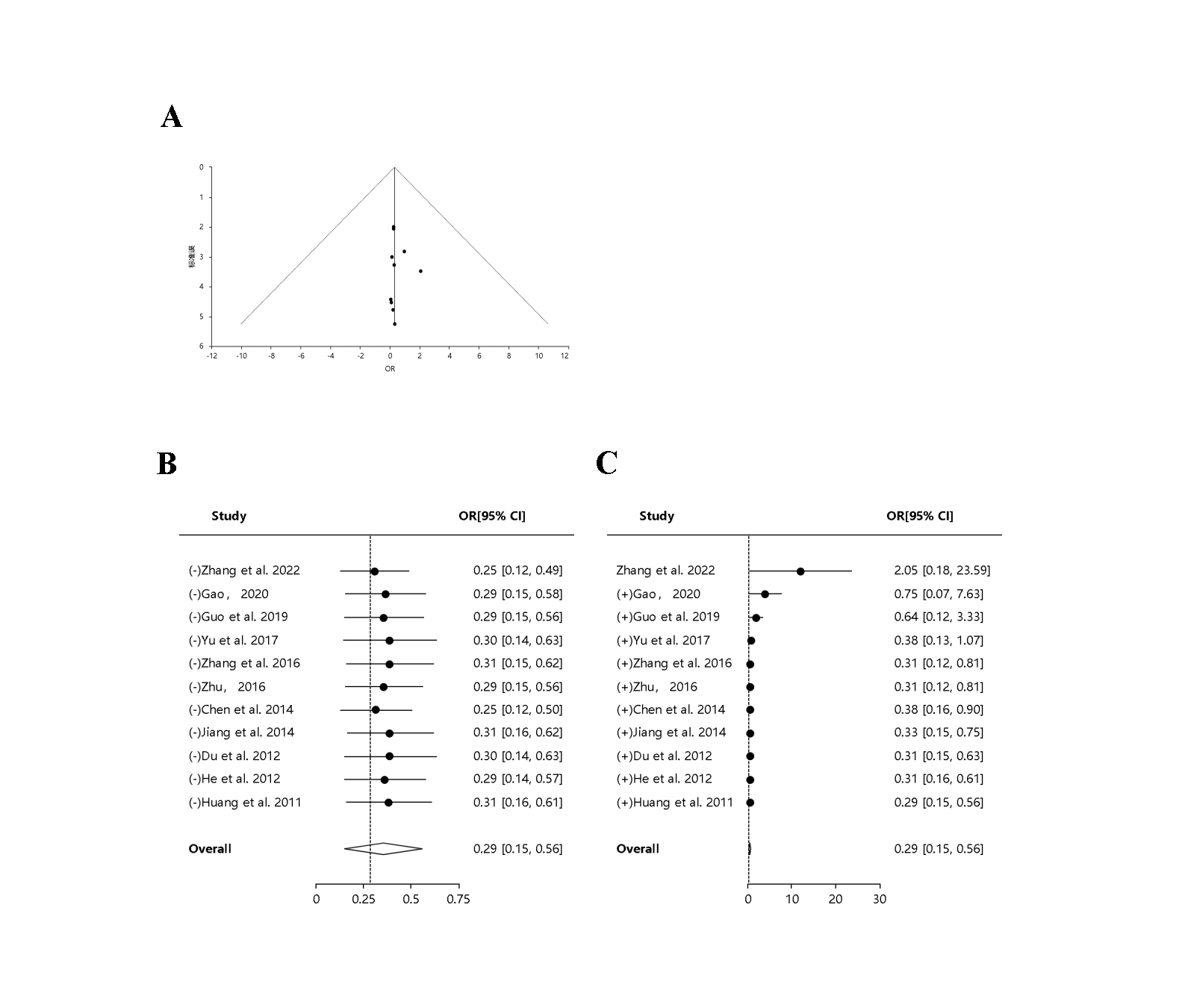


# Supplementary Figure S8. Publication bias, sensitivity analysis and meta-regression based on adverse events of clinical symptoms

(A) Funnel plots for publication bias of gastrointestinal reaction. (B) Sensitivity analysis of gastrointestinal reaction. (C) Meta-regression of gastrointestinal reaction. (D) Funnel plots for publication bias of cutaneous reaction. (E) Sensitivity analysis of cutaneous reaction. (F) Meta-regression of cutaneous reaction.


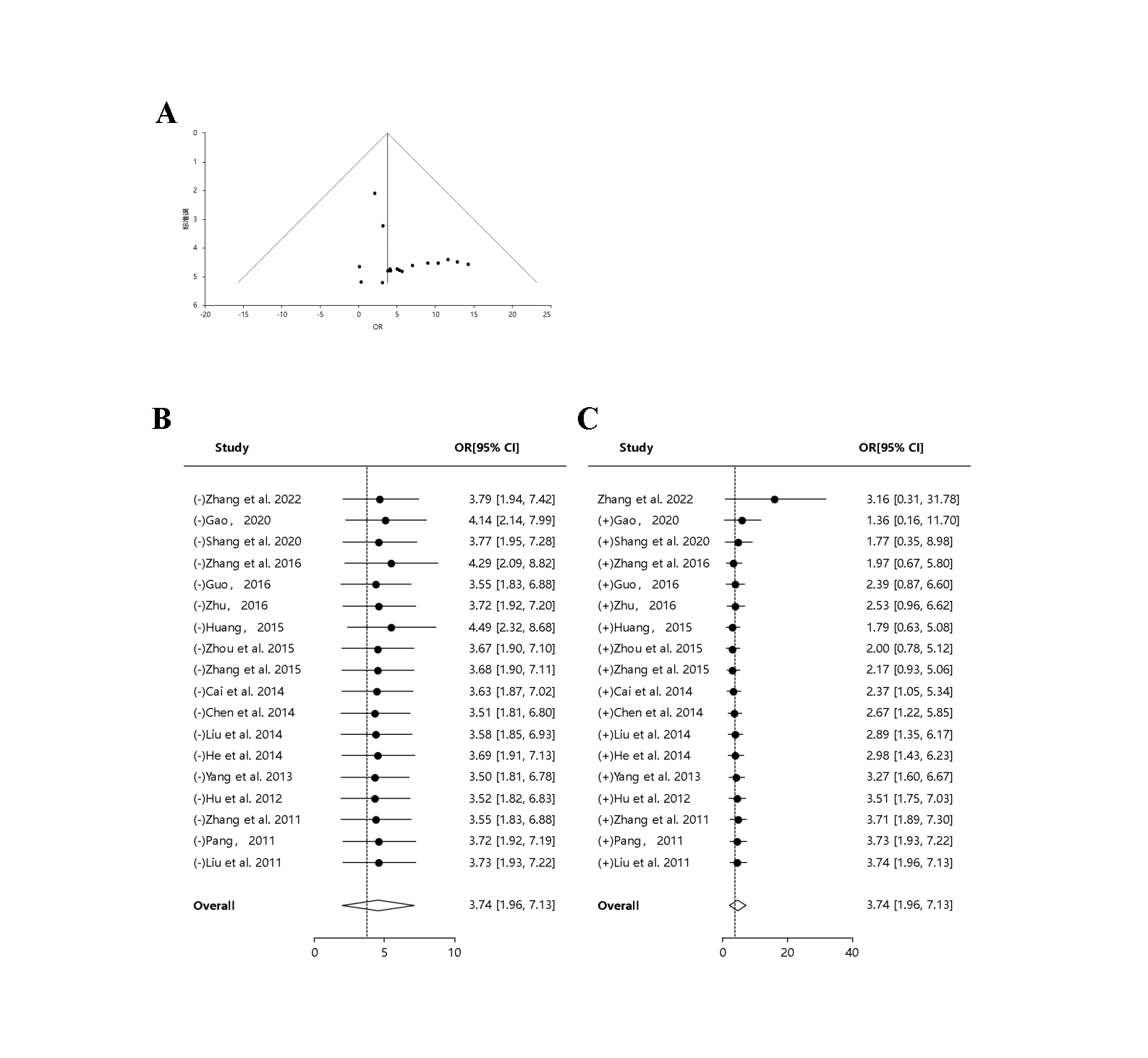


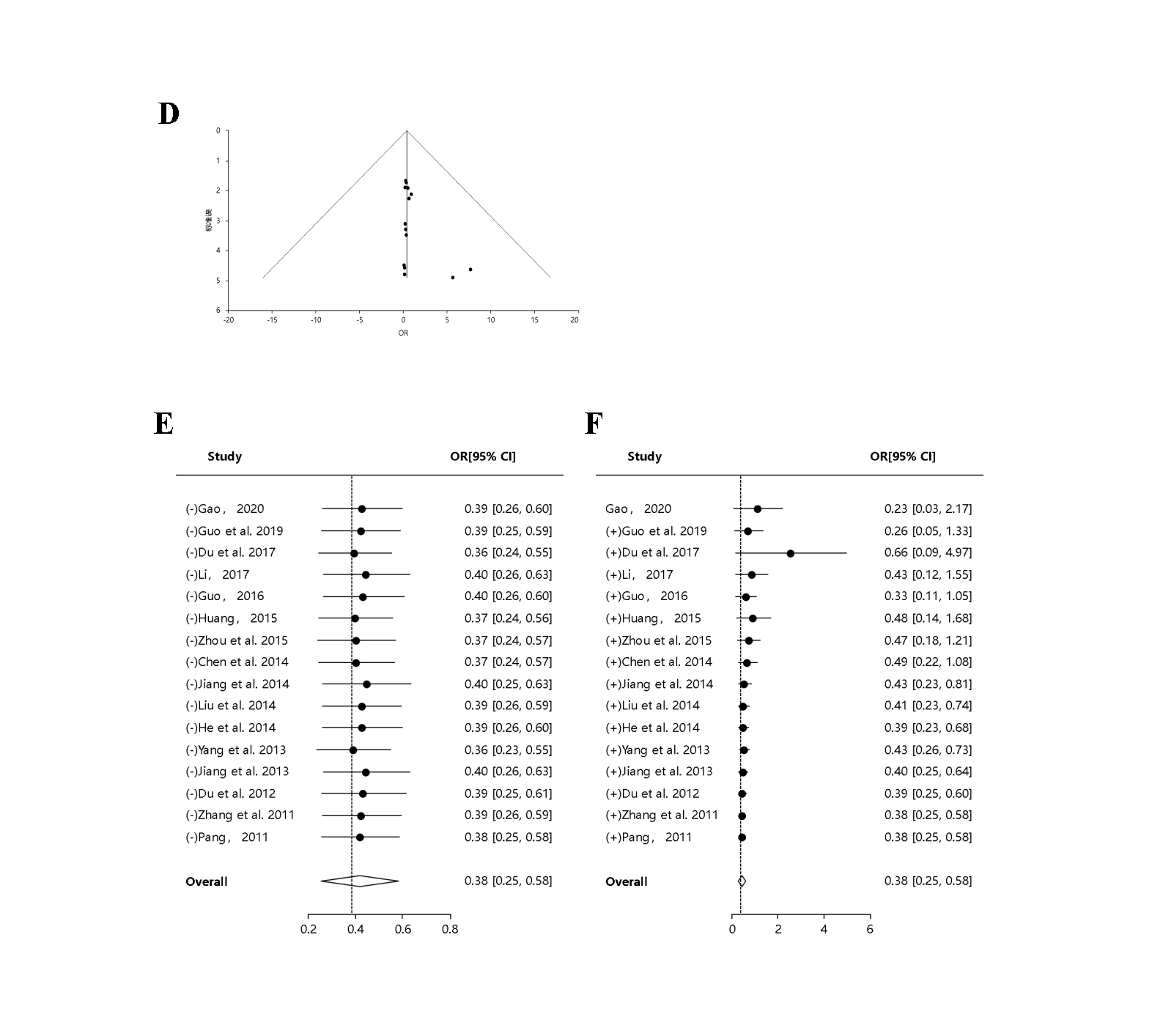


.

# Supplementary Table

# Supplementary Table 1. Characteristics of included studies.

| **First author,**  **year** | **Country;**  **setting** | **Blinding; number of arms** | **Treatment duration; Follow-up duration** | **Type of psoriasis** | **Severity of psoriasis** | **Duration of**  **psoriasis: mean (SD)** | **No. of I**  **/C; dropouts** | **Age: mean (SD); gender: M/F** | **Outcome measure** |
| --- | --- | --- | --- | --- | --- | --- | --- | --- | --- |
| Zhang et al. 2022 | China; hospital inpatients | NS; 2 | 12 w; 0 | Psoriasis vulgaris | Severe | I: 16.7 (3.2) m  C: 16.8 (3.8) m | I: 40/40; 0  C: 40/40; 0 | I: 35.21(5.28); 29/11  C: 34.92(5.60); 30/10 | ⑤⑥ |
| Gao, 2020 | China; hospital inpatients | NS; 2 | 8 w; 0 | NS | NS | I: 4.32 (0.72) y  C:4.29 (0.63) y | I: 47/47; 0  C: 47/47; 0 | I: 43.57(2.04); 26/21  C: 43.92(2.01); 24/23 | ⑤⑥ |
| Shang et al. 2020 | China; hospital inpatients | NS; 2 | 8 w; 0 | Psoriasis vulgaris | NS | I: 5.37 (0.23) y  C: 5.21 (0.18) y | I: 30/30; 0  C: 30/30; 0 | I: 34.6(4.2); 17/13  C: 35.7(5.3); 16/14 | ②⑥ |
| Ding, 2020 | China; hospital inpatients | NS; 2 | 4 w; 0 | NS | NS | I: 6.4 (2.6) y  C: 6.8 (2.3) y | I: 39/39; 0  C: 39/39; 0 | I: 29.2(6.4); 14/25  C: 28.7(6.4); 16/23 | ⑤ |
| Lv, 2020 | China; hospital inpatients | NS; 2 | 8 w; 0 | Psoriasis vulgaris | NS | I: 7.63 (1.83) y  C: 7.79 (1.72) y | I: 56/56; 0  C: 56/56; 0 | I: 44.58(7.12); 31/25  C: 45.01(6.93); 29/27 | ②④ |
| Guo et al. 2019 | China; hospital inpatients | NS; 2 | 8 w; 0 | NS | NS | I: 3.87 (1.72) y  C: 4.23 (1.68) y | I: 23/23; 0  C: 23/23; 0 | I:44.32(5.86); 14/10  C: 43.28(5.94); 13/10 | ②⑤⑥ |
| Zhang et al. 2018 | China; hospital inpatients | NS; 2 | 8 w; 24 w | Psoriasis vulgaris | NS | I: 6.74 (1.43) y  C: 6.62 (1.40) y | I: 50/50; 0  C: 50/50; 0 | I: 44.12(5.77); 35/15  C: 44.19(5.80); 33/17 | ③⑤ |
| Yu et al. 2017 | China; hospital outpatients | NS; 2 | 12 w; 0 | Psoriasis vulgaris | Moderate and severe | I: 138.62 (120.61) m  C: 158.3 (103.12) m | I: 53/40; 13  C: 55/44; 11 | I: 38.43(12.06); 35/18  C: 38.11(12.05); 36/19 | ①③⑥ |
| Song et al. 2017 | China; hospital inpatients | NS; 2 | 8 w; 0 | Psoriasis vulgaris | NS | I: 127.68 (96.75) m  C: 129.62 (98.62) m | I: 63/63; 0  C: 63/63; 0 | I: 43.52(12.03); 48/15  C: 43.29(11.76); 49/14 | ②④⑤ |
| Lin, 2017 | China; NS | NS; 2 | 8 w; 0 | Psoriasis vulgaris | Moderate | I: 18.81 (5.33) y  C: 19.74 (6.21) y | I: 67/67; 0  C: 62/62; 0 | I: 39.28(10.21); 39/28  C: 40.18(11.83); 37/25 | ⑤ |
| Du et al. 2017 | China; hospital outpatients | NS; 2 | 4 w; 0 | Psoriasis vulgaris | NS | NS | I: 32/32; 0  C: 32/32; 0 | I: 37.3(5.2); 21/11  C: 32.2(5.4); 18/14 | ⑥ |
| Li, 2017 | China; hospital outpatients | NS; 2 | 8 w; 0 | Psoriasis vulgaris | NS | I: 61.47 (22.53) m  C: 62.18 (24.25) m | I: 37/37; 0  C: 37/37; 0 | I: 37.15(9.24); 22/15  C: 36.52(9.16); 24/13 | ②⑥ |

# Supplementary Table 1. (Continued) Characteristics of included studies.

| **First author,**  **year** | **Country;**  **setting** | **Blinding; number of arms** | **Treatment duration; Follow-up duration** | **Type of psoriasis** | **Severity of psoriasis** | **Duration of**  **psoriasis: mean (SD)** | **No. of I**  **/C; dropouts** | **Age: mean (SD); gender: M/F** | **Outcome measure** |
| --- | --- | --- | --- | --- | --- | --- | --- | --- | --- |
| Li et al. 2016 | China; hospital outpatients | NS; 2 | 8 w; 0 | Psoriasis vulgaris | NS | I: 6.16 (4.29) y  C: 7.16 (4.98) y | I: 30/27; 3  C: 30/28; 2 | I: 45.23 (15.52); 19/11  C: 42.51 (9.52); 14/16 | ② |
| Wang, 2016 | China; NS | NS; 2 | NS; NS | Psoriasis vulgaris | NS | NS | I: 30/30; 0  C: 30/30; 0 | I: 39.87 (5.24); 16/14  C: 41.64 (4.38); 17/13 | ② |
| Zhang et al. 2016 | China; hospital inpatients | NS; 2 | 12 w; 0 | Psoriasis vulgaris | NS | I: 13.6 (3.9) y  C: 12.4 (3.5) y | I: 48/48; 0  C: 48/48; 0 | I: 36.8 (5.3); 30/18  C: 36.2 (5.1); 28/20 | ②④⑥ |
| Guo, 2016 | China; hospital inpatients | NS; 2 | 8 w; 0 | Psoriasis vulgaris | NS | NS | I:30/30; 0  C: 30/30; 0 | I: 36.4 (6.7); 19/11  C: 36.1 (6.4); 18/12 | ⑥ |
| Zhu, 2016 | China; hospital out/inpatients | NS; 4 | 6 w; 0 | Psoriasis vulgaris | NS | NS | I1: 11/11; 0  I2: 11/11; 0  C: 11/11; 0 | I1: 35.5 (10.4); 4/7  I2: 34.4 (10.0); 7/4  C: 36.6 (10.2); 7/4 | ②⑤⑥ |
| Huang, 2015 | China; NS | NS; 3 | 24 w; 0 | Psoriasis vulgaris | Moderate and severe | NS | I: 16/16; 0  C: 16/16; 0 | 35.2 (10.8); 26/22 | ②⑥ |
| Zou et al. 2015 | China; NS | NS; 3 | 12 w; 24w | Psoriasis vulgaris | NS | NS | I: 25/25; 0  C: 26/26; 0 | 37.75 (5.61); 42/34 | ②⑥ |
| Zhang et al. 2015 | China; NS | NS; 2 | 24w; 0 | Psoriasis vulgaris | Mild to moderate | I: 5.1 y  C: 4.9 y | I:48/45; 3  C: 48/46; 2 | I: 38.6; 22/26  C: 41.2; 20/28 | ①⑥ |
| Cai et al. 2014 | China; hospital outpatients | NS; 2 | 12 w; 0 | Psoriasis vulgaris | NS | NS | I: 38/38; 0  C: 35/35; 0 | I: 43.66 (13.35); 24/14  C: 45.40 (12.26); 22/13 | ③⑥ |
| Chen et al. 2014 | China; hospital out/inpatients | NS; 2 | 8 w; 0 | Psoriasis vulgaris | Moderate and severe | I: 6.35 (3.24) y  C: 6.86 (4.03) y | I: 30/28; 2  C: 30/27; 3 | I: 40.12 (13.23);16/12  C: 42.35 (12.25); 14/13 | ②⑥ |
| Jiang et al. 2014 | China; hospital out/inpatients | NS; 2 | 12 w; 0 | Psoriasis vulgaris | Moderate and severe | I: 5.4 (2.3) y  C: 5.7 (2.5) y | I: 35/35; 0  C: 34/34; 0 | I: 33.9 (12.4); 20/15  C: 34.4 (13.7); 19/15 | ②⑥ |
| Liu et al. 2014 | China; hospital outpatients | NS; 3 | 6 w; 12w | Psoriasis vulgaris | NS | I: 6.25 (3.23) y  C: 5.89 (2.97) y | I: 36/36; 0  C: 32/32; 0 | I: 39.15 (13.68); 18/18  C: 41.71 (12.93); 17/15 | ②⑥ |

# Supplementary Table 1. (Continued) Characteristics of included studies.

| **First author,**  **year** | **Country;**  **setting** | **Blinding; number of arms** | **Treatment duration; Follow-up duration** | **Type of psoriasis** | **Severity of psoriasis** | **Duration of**  **psoriasis: mean (SD)** | **No. of I**  **/C; dropouts** | **Age: mean (SD); gender: M/F** | **Outcome measure** |
| --- | --- | --- | --- | --- | --- | --- | --- | --- | --- |
| He et al. 2014 | China; hospital outpatients | NS; 2 | 8 w; 0 | Psoriasis vulgaris | Moderate | NS | I: 78/78; 0  C: 76/76; 0 | NS | ②⑥ |
| Yang et al. 2013 | China; hospital outpatients | NS; 2 | 8 w; 12w | Psoriasis vulgaris | NS | I: 69.7 (62.5) m  C: 67.4 (63.3) m | I: 34/34; 0  C: 34/34; 0 | I: 35.74 (11.53); 18/16  C: 33.89 (12.78); 17/17 | ②⑥ |
| Jiang et al. 2013 | China; hospital out/inpatients | NS; 2 | 6 w; 0 | Psoriasis vulgaris | NS | 15.48 (12.25) y | I: 38/38; 0  C: 24/24; 0 | 34.33 (16.45); 40/22 | ②⑥ |
| Hu et al. 2012 | China; hospital out/inpatients | NS; 2 | 6 w; 0 | Psoriasis vulgaris | NS | I: 11.6 (3.5) y  C: 12.3 (2.7) y | I:50/50; 0  C:50/50; 0 | I: 37.3 (11.5); 27/13  C: 35.8 (12.3); 28/22 | ②⑤ |
| Du et al. 2012 | China; hospital out/inpatients | NS; 2 | 12 w; 0 | Pustular psoriasis | NS | I: 7 (4.7) y  C: 6 (5.1) y | I: 31/31; 0  C: 30/30; 0 | I: 41 (5.2); 17/14  C: 43 (6,2); 18/12 | ②⑥ |
| He et al. 2012 | China; hospital outpatients | NS; 2 | 8 w; 0 | NS | Moderate and severe | I: 5.23 (3.23) y  C: 5.09 (3.76) y | I: 32/32; 0  C: 30/30; 0 | I: 41.23 (14.17); 18/14  C: 40.18 (11.36); 16/14 | ②⑥ |
| Hua et al. 2012 | China; hospital outpatients | NS; 2 | 8 w; 0 | Psoriasis vulgaris | NS | I: 42.96 (25.23) m  C: 52.65 (11.20) m | I: 23/23; 0  C: 23/23; 0 | I: 55.35 (10.92); 16/7  C: 52.65 (11.20); 14/9 | ② |
| Hu et al. 2012 | China; hospital patients (NS) | NS; 2 | 12 w; 0 | Psoriasis vulgaris | NS | I: 3.75y  C: 3.75y | I: 90/90; 0  C: 90/90; 0 | I: 37.5 (12.2); 48/42  C: 38.5 (11.5); 46/44 | ②⑥ |
| Zhang et al. 2011 | China; hospital outpatients | NS; 3 | 6 w; 0 | Psoriasis vulgaris | NS | I: 67.3 (59.8) m  C: 68.3 (67.2) m | I: 30/30; 0  C: 30/30; 0 | I: 35.6 (10.4); 18/12  C: 38.5 (9.8); 16/14 | ②⑥ |
| Pang, 2011 | China; hospital patients (NS) | NS; 2 | 8 w; 0 | Psoriasis vulgaris | NS | I: 8.32 (4.61) y  C: 5.26 (3.57) y | I: 38/38; 0  C: 30/30; 0 | I: 23.41 (6.18); 17/21  C: 27.41 (7.12); 18/12 | ②⑥ |
| Huang et al. 2011 | China; hospital patients (NS) | NS; 2 | 8 w; 0 | Psoriasis vulgaris | NS | I: 3.6 y  C: 2.8 y | I: 39/39; 0  C: 32/32; 0 | I: 24.8; 25/14  C: 23.5; 23/9 | ②⑥ |
| Liu et al. 2011 | China; hospital outpatients | NS; 3 | 4 w; 0 | Psoriasis vulgaris | NS | NS | I: 36/36; 0  C: 26/26; 0 | 38.1 (9.6); 46/44 | ②⑥ |

TGP, total glucosides of paeony; C, control; I, intervention; AC, acitretin capsule; NB-UVB, narrow-band UVB; NS, not stated; PASI, Psoriasis Area and Severity Index; w, weeks; m, months; y, years; M, male; F, female; ① PASI 75; ② PASI 60; ③ PASI 50; ④ BSA; ⑤ inflammatory factors; ⑥ adverse events.

# Supplementary Table 2. Detailed information concerning the intervention/comparators of included studies.

| **First author,**  **year** | **Intervention (TGP+conventional therapy)** | **Comparators (conventional therapy)** | **TGP preparation type and dosage** | **Conventional therapy dosage and administration** |
| --- | --- | --- | --- | --- |
| Zhang et al. 2022 | TGP + Etanercept | Etanercept | NS, 0.6g po tid | 25 mg sc. twice a week |
| Gao, 2020 | TGP + AC | Acitretin capsule | Capsule, 0.6g po bid | 25 mg/d as initial oral dosage, adjusted dosage based on severity and medication tolerance |
| Shang et al. 2020 | TGP + Desonide cream | Desonide cream | Capsule, 0.6g po tid | Topical use, bid |
| Ding, 2020 | TGP + NB-UVB, Tacrolimus ointment | Tacrolimus ointment, NB-UVB | Capsule, 0.6g po bid | Tacrolimus ointment, topical use, bid; NB-UVB, 3 days in a row |
| Lv, 2020 | TGP + AC + Compound flumetasone ointment | Acitretin capsule, Compound flumetasone ointment | Capsule, 0.6g po bid | Acitretin capsule, 25-30 mg/d as initial oral dosage, adjusted dosage based on severity and medication tolerance. The maximum dose does not exceed 75 mg/d; Compound flumetasone ointment, topical use, qd or bid |
| Guo et al. 2019 | TGP + Erythromycin ointment + Sulfur ointment | Erythromycin ointment, Sulfur ointment | Capsule, 0.6g po tid | Erythromycin ointment, topical use, qd; Sulfur ointment, topical use, tid |
| Zhang et al. 2018 | TGP + AC + NB-UVB | NB-UVB, Acitretin capsule | Capsule, 0.6g po tid | NB-UVB, three times a week; Acitretin capsule, 20 mg/d for body mass> 70 kg |
| Yu et al. 2017 | TGP + AC | Acitretin capsule | Capsule, 0.6g po bid in first week, then adjusted dosage to 0.6g po tid | A dose of 20 mg/day if the patient weighed less than or equal to 70 kg, or 30 mg/day if the patient weighed greater than 70 kg |
| Song et al. 2017 | TGP + AC + Compound flumetasone ointment | Acitretin capsule, Compound flumetasone ointment | Capsule, 0.6g po tid | Acitretin capsule,10 mg/d po; Compound flumetasone ointment, qd |
| Lin, 2017 | TGP + NB-UVB + Calcipotriol cream | NB-UVB, Calcipotriol cream | Capsule, 0.6g po tid | NB-UVB, three times a week; Calcipotriol cream, topical use, bid |
| Du et al. 2017 | TGP + Calcipotriol betamethasone ointment | Calcipotriol betamethasone ointment | Capsule, 0.6g po tid | Topical use, qd |
| Li, 2017 | TGP + NB-UVB | NB-UVB | Capsule, 0.6g po tid | qod |
| Li et al. 2016 | TGP + NB-UVB | NB-UVB | Capsule, 0.6g po tid | Three times a week |
| Wang, 2016 | TGP + Compound flumetasone ointment | Compound flumetasone ointment | Capsule, 0.6g po tid | Topical use, qd or bid |
| Zhang et al. 2016 | TGP + Methotrexate tablet + Vaseline | Methotrexate tablet, vaseline | Capsule, 0.6g po tid | Methotrexate tablet, 2.5 mg po, three times a week; Vaseline |
| Guo, 2016 | TGP + NB-UVB | NB-UVB | Capsule, 0.6g po tid | Three times a week |
| Zhu, 2016 | I1: TGP + urea ointment  I2: TGP+ urea ointment | Urea ointment | I1: Capsule, 0.6g po tid  I2: Capsule, 0.6g po bid | Topical use, bid |
| Huang, 2015 | TGP + Methotrexate tablet | Methotrexate tablet | Capsule, 0.4-0.6 g/d | 7.5 mg po, once a week |

# Supplementary Table 2. (Continued) Detailed information concerning the intervention/comparators of included studies.

| **First author,**  **year** | **Intervention (TGP+conventional therapy)** | **Comparators (conventional therapy)** | **TGP preparation type and dosage** | **Conventional therapy dosage and administration** |
| --- | --- | --- | --- | --- |
| Zou et al. 2015 | TGP + NB-UVB | NB-UVB | Capsule, 0.6g po tid | Three times a week |
| Zhang et al. 2015 | TGP + NB-UVB | NB-UVB | Capsule, 0.6g po tid | Once every three days |
| Cai et al. 2014 | TGP + Calcipotriol ointment | 0.005% Calcipotriol Ointment | Capsule, 0.6g po tid | Topical use, bid |
| Chen et al. 2014 | TGP + NB-UVB + AC | NB-UVB, Acitretin capsule | Capsule, 0.6g po tid | NB-UVB, three times a week; Acitretin capusle, 0.5 mg/(kg·d) |
| Jiang et al. 2014 | TGP + AC + Desonide cream | Acitretin capsule, Desonide cream | Capsule, 0.6g po tid | Acitretin capsule, 30 - 60 mg/d as initial oral dosage, adjusted dosage based on condition severity and medication tolerance, 10–30 mg/d as maintenance dosage; Desonide cream, topical use, bid |
| Liu et al. 2014 | TGP + Tretinoin ointment + Mometasone furoate cream | 0.01% Tretinoin ointment, Mometasone furoate cream | Capsule, 0.6g po tid | 0.01% Tretinoin ointment, topical use, qd; Mometasone furoate cream, topical use, qd |
| He et al. 2014 | TGP + NB-UVB + Calcipotriol cream | NB-UVB, Calcipotriol cream | Capsule, 0.6g po tid | NB-UVB, qod; Calcipotriol cream, topical use, bid |
| Yang et al. 2013 | TGP + NB-UVB | NB-UVB | Capsule, 0.6g po tid | Three times a week |
| Jiang et al. 2013 | TGP + NB-UVB | NB-UVB | Capsule, 0.6g po tid | NS |
| Hu et al. 2012 | TGP + NB-UVB | NB-UVB | Capsule, 0.6g po tid | qod |
| Du et al. 2012 | TGP + AC + Hydrocortisone butyrate cream | Acitretin, Hydrocortisone butyrate cream, Skin care oil | Capsule, 0.6g po tid | Acitretin capsule, 30 - 60 mg/d; Hydrocortisone butyrate cream, Skin care oil, topical use, bid |
| He et al. 2012 | TGP + AC | Acitretin capsule | Capsule, 0.6g po tid | 0.5mg/(kg·d) po |
| Hua et al. 2012 | TGP + Compound glycyrrhizin tablet | Compound glycyrrhizin tablet | Capsule, 0.6g po bid | 75 mg po tid |
| Hu et al. 2012 | TGP + Tripterygium tablet + Hhydrocortisone ointment | Tripterygium tablet, Hydrocortisone ointment | Capsule, 0.6g po tid | Tripterygium tablet, 20 mg po bid; Hydrocortisone ointment |
| Zhang et al. 2011 | TGP + 5% Pine tar ointment | 5% Pine tar ointment | Capsule, 0.6g po tid | Topical use, bid |
| Pang, 2011 | TGP + NB-UVB + Compound flumetasone ointment | NB-UVB, Compound flumetasone ointment | Capsule, 0.6g po tid | NB-UVB, qod; Compound flumetasone ointment, topical use bid |
| Huang et al. 2011 | TGP + AC + Non glucocorticoids | Acitretin capsule, Compound flumetasone ointment | Capsule, 0.6g po tid | Acitretin capsule,10 mg po tid; non glucocorticoids |
| Liu et al. 2011 | TGP + compound glycyrrhizin injection | Compound glycyrrhizin injection | Capsule, 0.6g po tid | 80 mg/d ivgtt |

TGP, total glucosides of paeony; NB-UVB, narrow-band ultraviolet B radiation; NS, not stated; po, administered orally; qd, once daily; qod, once every two days; tid, bid, twice daily; three times daily. ivgtt, intravenously guttae.

# Supplementary File

# Supplementary File S1. The PRISMA checklist of this meta-analysis

| **Section and Topic** | **Item #** | **Checklist item** | **Location where item is reported** |
| --- | --- | --- | --- |
| **TITLE** | | |  |
| Title | 1 | Identify the report as a systematic review. | P1 |
| **ABSTRACT** | | |  |
| Abstract | 2 | See the PRISMA 2020 for Abstracts checklist. | P1-2 |
| **INTRODUCTION** | | |  |
| Rationale | 3 | Describe the rationale for the review in the context of existing knowledge. | P2 |
| Objectives | 4 | Provide an explicit statement of the objective(s) or question(s) the review addresses. | P2 |
| **METHODS** | | |  |
| Eligibility criteria | 5 | Specify the inclusion and exclusion criteria for the review and how studies were grouped for the syntheses. | P3 |
| Information sources | 6 | Specify all databases, registers, websites, organisations, reference lists and other sources searched or consulted to identify studies. Specify the date when each source was last searched or consulted. | P3 |
| Search strategy | 7 | Present the full search strategies for all databases, registers and websites, including any filters and limits used. | P3 |
| Selection process | 8 | Specify the methods used to decide whether a study met the inclusion criteria of the review, including how many reviewers screened each record and each report retrieved, whether they worked independently, and if applicable, details of automation tools used in the process. | P3 |
| Data collection process | 9 | Specify the methods used to collect data from reports, including how many reviewers collected data from each report, whether they worked independently, any processes for obtaining or confirming data from study investigators, and if applicable, details of automation tools used in the process. | P3 |
| Data items | 10a | List and define all outcomes for which data were sought. Specify whether all results that were compatible with each outcome domain in each study were sought (e.g. for all measures, time points, analyses), and if not, the methods used to decide which results to collect. | P3 |
|  | 10b | List and define all other variables for which data were sought (e.g. participant and intervention characteristics, funding sources). Describe any assumptions made about any missing or unclear information. | P3 |
| Study risk of bias assessment | 11 | Specify the methods used to assess risk of bias in the included studies, including details of the tool(s) used, how many reviewers assessed each study and whether they worked independently, and if applicable, details of automation tools used in the process. | P3 |
| Effect measures | 12 | Specify for each outcome the effect measure(s) (e.g. risk ratio, mean difference) used in the synthesis or presentation of results. | P4 |
| Synthesis methods | 13a | Describe the processes used to decide which studies were eligible for each synthesis (e.g. tabulating the study intervention characteristics and comparing against the planned groups for each synthesis (item #5)). | P4 |
|  | 13b | Describe any methods required to prepare the data for presentation or synthesis, such as handling of missing summary statistics, or data conversions. | P4 |
|  | 13c | Describe any methods used to tabulate or visually display results of individual studies and syntheses. | P4 |
|  | 13d | Describe any methods used to synthesize results and provide a rationale for the choice(s). If meta-analysis was performed, describe the model(s), method(s) to identify the presence and extent of statistical heterogeneity, and software package(s) used. | P4 |
|  | 13e | Describe any methods used to explore possible causes of heterogeneity among study results (e.g. subgroup analysis, meta-regression). | P4 |
|  | 13f | Describe any sensitivity analyses conducted to assess robustness of the synthesized results. | P4 |
| Reporting bias assessment | 14 | Describe any methods used to assess risk of bias due to missing results in a synthesis (arising from reporting biases). | P4 |
| Certainty assessment | 15 | Describe any methods used to assess certainty (or confidence) in the body of evidence for an outcome. | P4 |
| **RESULTS** | | |  |
| Study selection | 16a | Describe the results of the search and selection process, from the number of records identified in the search to the number of studies included in the review, ideally using a flow diagram. | P5 and Figure 1 |
|  | 16b | Cite studies that might appear to meet the inclusion criteria, but which were excluded, and explain why they were excluded. | P5 and Figure 1 |
| Study characteristics | 17 | Cite each included study and present its characteristics. | P5 and Table1 |
| Risk of bias in studies | 18 | Present assessments of risk of bias for each included study. | P2 and Figure 2-3 |
| Results of individual studies | 19 | For all outcomes, present, for each study: (a) summary statistics for each group (where appropriate) and (b) an effect estimate and its precision (e.g. confidence/credible interval), ideally using structured tables or plots. | P5-P10 and Figure4-11 |
| Results of syntheses | 20a | For each synthesis, briefly summarise the characteristics and risk of bias among contributing studies. | P5-P10 and Figure4-11 |
|  | 20b | Present results of all statistical syntheses conducted. If meta-analysis was done, present for each the summary estimate and its precision (e.g. confidence/credible interval) and measures of statistical heterogeneity. If comparing groups, describe the direction of the effect. | P5-P10 and Figure4-11 |
|  | 20c | Present results of all investigations of possible causes of heterogeneity among study results. | P5-P10 and supplementary figure S1-S8 |
|  | 20d | Present results of all sensitivity analyses conducted to assess the robustness of the synthesized results. | P5-P10 and supplementary figure S1-S8 |
| Reporting biases | 21 | Present assessments of risk of bias due to missing results (arising from reporting biases) for each synthesis assessed. | P5-P10 and supplementary figure S1-S8 |
| Certainty of evidence | 22 | Present assessments of certainty (or confidence) in the body of evidence for each outcome assessed. | P5-P10 and supplementary figure S1-S8 |
| **DISCUSSION** | | |  |
| Discussion | 23a | Provide a general interpretation of the results in the context of other evidence. | P10 |
|  | 23b | Discuss any limitations of the evidence included in the review. | P11 |
|  | 23c | Discuss any limitations of the review processes used. | P11 |
|  | 23d | Discuss implications of the results for practice, policy, and future research. | P12 |
| **OTHER INFORMATION** | | |  |
| Registration and protocol | 24a | Provide registration information for the review, including register name and registration number, or state that the review was not registered. | P2 |
|  | 24b | Indicate where the review protocol can be accessed, or state that a protocol was not prepared. | P2 |
|  | 24c | Describe and explain any amendments to information provided at registration or in the protocol. | P2 |
| Support | 25 | Describe sources of financial or non-financial support for the review, and the role of the funders or sponsors in the review. | P12 |
| Competing interests | 26 | Declare any competing interests of review authors. | P12 |
| Availability of data, code and other materials | 27 | Report which of the following are publicly available and where they can be found: template data collection forms; data extracted from included studies; data used for all analyses; analytic code; any other materials used in the review. | P12 |

*From:*  Page MJ, McKenzie JE, Bossuyt PM, Boutron I, Hoffmann TC, Mulrow CD, et al. The PRISMA 2020 statement: an updated guideline for reporting systematic reviews. BMJ 2021;372:n71. doi: 10.1136/bmj.n71

For more information, visit: <http://www.prisma-statement.org/>
